# Pediatric Mini-Tablets: Predicting the Hidden Risk of Fill Errors

**DOI:** 10.3390/pharmaceutics15020594

**Published:** 2023-02-10

**Authors:** Brandon G. Gerberich, Grace A. Okoh, James C. DiNunzio, Michael B. Lowinger

**Affiliations:** Oral Formulation Sciences, Merck & Co., Inc., Kenilworth, NJ 07065, USA

**Keywords:** mini-tablet, content uniformity, fill count, sachet, fill errors, dose uniformity

## Abstract

Compressed mini-tablets in sachets or capsules are an increasingly prevalent oral solid dosage form for pediatric products. While resembling adult tablets, additional care is required to control weight and potency (blend uniformity) variation due to their small size (≤2.5 mm average diameter). Additionally, sachet fill count errors complicate dose accuracy as they are difficult to resolve with weight-checking equipment. This study quantified the probability of failing content uniformity (CU) specifications (which results in the inability to release a batch) defined in USP <905> using a Monte Carlo computational model. Failure risk was modeled as a function of sachet fill count, mini-tablet weight, potency distribution, and fill error frequency. The model allows product developers to (1) determine appropriate fill counts based on anticipated product weight and potency relative standard deviation (RSD), (2) set fill error probability tolerances for sachet filling processes, (3) identify CU improvement opportunities, and (4) quantify the probability of CU failure informing risk management activities and risk disclosure for regulatory agencies. A representative product with weight and potency RSD no greater than 5%, fill count of 1–4 mini-tablets per sachet, and fill error probability per mini-tablet filled of 0.1% may experience CU batch failure probabilities as high as 8.23%, but only 0.283% if the fill count is increased to 5–10 mini-tablets per sachet. Generally, fill counts of less than five mini-tablets per sachet should be avoided where possible.

## 1. Introduction

**Figure 1:**
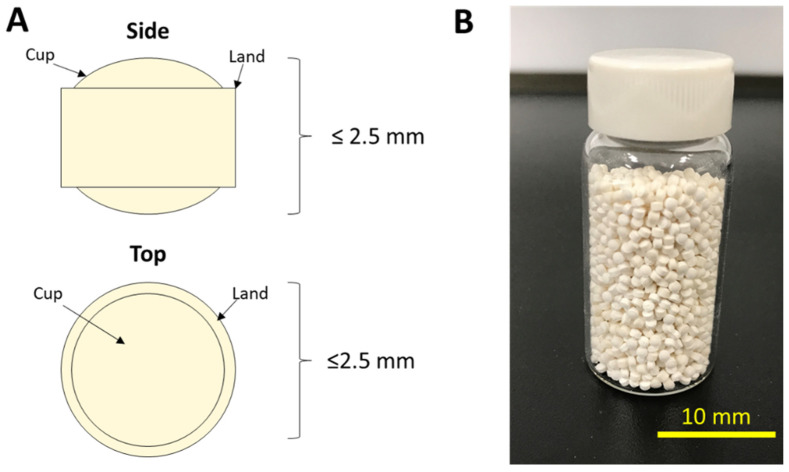
Oral granules comprise a compressed oral solid dosage form 1.0–2.5 mm in diameter and offer a quasi-flexible dosing solution for pediatric populations by varying fill count per dosage unit. (**A**) schematic illustration of mini-tablet features and (**B**) image of a vial filled with mini-tablets 2 mm in diameter (therefore qualifying as oral granules and treated as such for content uniformity evaluation).

Pediatric formulation development is undergoing transformation due to recent changes in regulatory policies promoting the advancement of age-appropriate medicines [1,2]. As a result, age-appropriate dosage forms have been developed to meet the increased emphasis on pediatric care [3]. One particular dosage form allowing for flexible dosing is pediatric mini-tablets (Figure 1) filled into sachets or capsules [4,5]. When less than 2.5 mm in average diameter, they are classified as oral granules by the Food and Drug Administration (FDA) [6]. As such, multiple mini-tablets may be combined in a single dosage unit to achieve the desired dose, thus enabling personalized therapeutics even beyond pediatric populations. However, small size also increases weight and potency variance, increasing the risk of failing content uniformity (CU) specifications [7]. This risk is additionally complicated by the potential for fill count errors while filling mini-tablets into sachets or capsules (the term “sachets” will be used in this publication) for small dosage (low fill count) units.

Testing requirements ensure drug product quality and patient safety. Batches with significant variance in drug content may be rejected due to CU failure. In the United States, CU specifications are outlined in USP <905>, the specific rules for which are determined by the dosage form, potency, and drug load of the solid dosage form in question [8]. Mini-tablet unit doses with ≥25% drug load and ≥25 mg potency is subject to weight variation tests, while those with <25% drug load or <25 mg potency are subject to CU testing. Importantly, each sachet is considered one unit dose. In this study, the probability of failing USP <905> for small fill count sachets (≤10) was examined, which often falls under the CU specification. Though the model accounts for the USP <905> CU specification, it is also valid for weight variation since it represents the simplified scenario where potency variation is assumed to be 0. 

Fill count errors result in large dose variations during the final counting/packaging step when the total count is low. Unfortunately, these errors are often not resolvable by weight-checking equipment because either (1) sachet weight variance regularly exceeds that of a single mini-tablet, or (2) the weight of a single mini-tablet is less than the weight checker’s limit of detection. Fill error probability depends on equipment maintenance, setup, and product specifications. The passage of each mini-tablet into the sachet can be considered an independent event where a miscount may occur. Miscounts may be either overfills, such as when an equipment sensor fails to resolve two mini-tablets passing by, or underfills, such as when an equipment sensor produces a false positive reading.

The cause and impact of fill count errors are varied and depend on the equipment used and desired fill count. This is complicated by the variety of filling equipment designs available [9]. Common causes include suboptimal settings of the mini-tablet dosing mechanism, buildup/blockage of mini-tablets in the feeding chute, poorly tuned sensor sensitivity, and dust accumulation on sensors. Because low-frequency errors are difficult to detect, there is a need to understand their impact on product quality specifications. Mini-tablet sampling/testing strategies for product release have been the subject of a recent discussion with consideration for the large testing burden required, highlighting the need for a clearer understanding of dose variation risks [10]. In low fill count products, a single error results in an average dose deviation equal to the inverse of fill count [7].

The importance of dose accuracy of mini-tablet products has been reflected in recent investigations. Previous studies have employed the Monte Carlo simulation technique to study the impact of attributes such as particle size distribution on CU, though these did not consider fill errors [11,12]. Related to particle size, the percolation threshold impacts dissolution and tensile strength and is associated with potency [13,14]. Others have considered the theoretical impact of fill errors as a function of fill count and measured CU experimentally, rather than computationally, for ranges of mini-tablet weight and potency variance [7,15]. Monte Carlo simulations have even been used to model the impacts of pellet (similar to mini-tablets) shape, size, and distribution when filled into capsules to inform on filling accuracy [16,17]. 

In this study, a Monte Carlo simulation was created to calculate the probability of batch failure according to USP <905> CU specifications. Two sources of variance were included in the model: (1) normally distributed assay of individual mini-tablets due to weight or potency heterogeneity, and (2) Bernoulli-distributed discrete fill errors during the sachet filling process resulting in some sachets with unintended fill counts. Key product parameters, therefore, included (1) weight variance, (2) potency variance, (3) sachet fill count, and (4) fill error probability which has not been modeled in combination previously. Of particular interest is the impact of fill error probability on content uniformity. The model results are intended to inform pediatric mini-tablet product development decisions.

## 2. Methods

### 2.1. Model Parameters

A Monte Carlo simulation was implemented in MATLAB 2019b (Natick, MA, USA: The MathWorks Inc.). The model predicts the probability of USP <905> stage 1 and stage 2 batch failure using inputs of weight relative standard deviation (RSD), potency RSD, sachet fill count, and fill error probability (on a per-mini-tablet basis). Note bulk mini-tablet mean weight and mean potency were assumed to be, on average, 100% of target weight and potency. The model calculations are, therefore, independent of the absolute weight and potency used. Potency RSD is defined here as the RSD of active drug substance content in a mini-tablet normalized by mini-tablet weight and may be described as “blend uniformity”. Fill error probability is defined as the probability of a single miscount error occurring for each mini-tablet filled into a sachet (not the probability that any given sachet would have a miscount error). Multiple miscounts were allowed per mini-tablet according to the Bernoulli distribution. See Supplementary Materials Sections S1.1–S1.3 for model design. 

### 2.2. Application of USP <905> Criteria to Statistical Model

**Figure 2:**
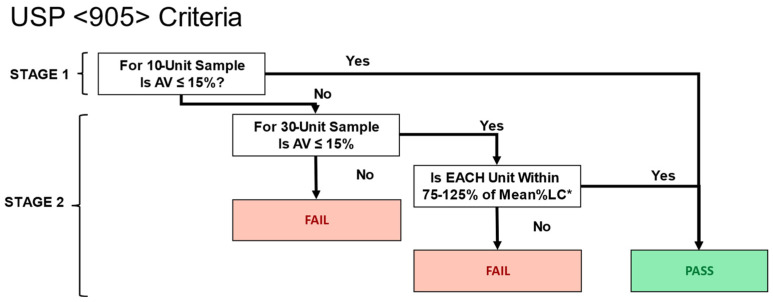
Simplified representation of USP <905> model decision matrix for CU assessment. In the Stage 1 assessment, the contents of 10 individual sachets are assayed to calculate an acceptance value (AV). If the AV is less than 15%, the batch passes CU testing. However, if the AV is greater than or equal to 15%, Stage 2 testing is required. In Stage 2 testing, the contents of additional 20 sachets are assayed, and data are aggregated with the initial ten sachets from stage 1. As in stage 1, an AV is calculated and compared to the 15% threshold. If AV is greater than 15% in Stage 2, the batch fails CU testing. If it passes, an additional criterion is required for the contents of individual sachets. Here, the contents of each sachet must be within plus/minus 25% of the target label claim. Failure to meet this criterion results in batch failure, and success results in batch passage of CU criteria. * Note, this criterion represents the case where the mean %LC is 98.5–101.5% of the target. It varies slightly if the mean %LC is outside this range and may be as low as 73.875–123.125% and as high as 76.125–126.875%.

Passage or failure of USP <905> is determined in two stages (Figure 2). In stage 1, a sample of 10 sachets is selected, and the contents of each are separately tested for weight and assay. From these, an Acceptance Value (AV) derived from the USP <905> criteria is calculated and compared against a reference value (AV must be ≤15%). Failure to meet the reference value triggers a second stage (stage 2) of testing which includes a sampling of 20 sachets in addition to the original 10. A second AV value is calculated for stage 2 and compared to the reference value (AV must be ≤15%). Failure to meet the reference criteria results in stage 2 failure and, consequently, failure to release the batch. Additionally, during stage 2 testing only, all individual doses must be within 25% of the target content for the batch to pass testing. 

Sachets were grouped into sets of 30, from which USP <905> parameters, including assay, were calculated to derive acceptance values (AV). This included an initial subset of 10 sachets for stage 1 calculations and a total of 30 sachets for stage 2 calculations. AVs were checked against the USP <905> criteria in silico to determine the passage or failure of each simulated set of sachets. The probability of failure was calculated as the frequency of failures divided by the number of sets of sachets simulated. USP <905> criteria for CU, rather than weight variation, was used because at low fill counts, doses commonly fall under 25 mg or 25% drug load threshold specified in USP <905>. 

### 2.3. Simplification of Weight and Potency RSD Parameters

**Figure 3:**
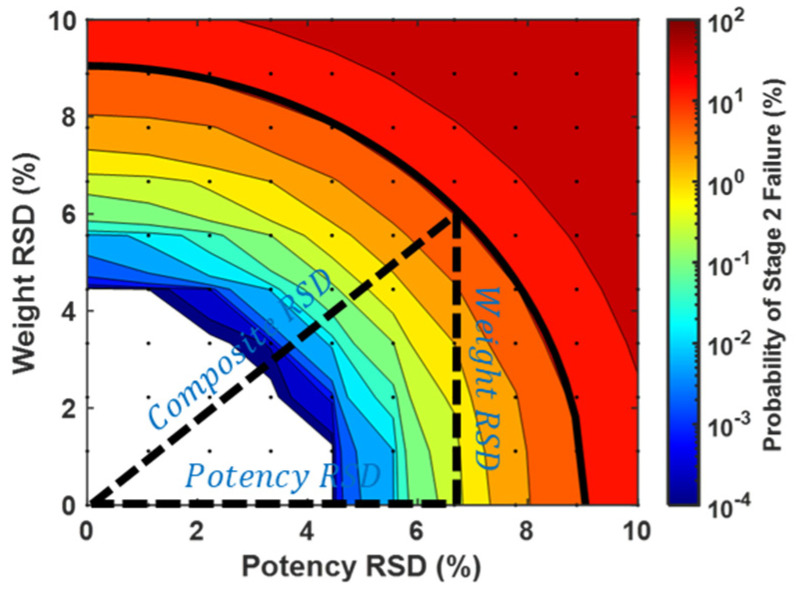
The probability of failing CU requirements was found to be qualitatively proportional to the root sum of squares of bulk mini-tablet weight RSD and potency RSD (Equation (1)). The composite RSD (bold solid line) is overlaid on the simulated probability of stage 2 failure with a fill count equal to 1 and a fill count probability equal to 0%. A similar trend is observed for other fill counts as well (Figure 4).

To reduce the number of parameters simulated, the weight and potency variances were combined into a single variance parameter—the “Composite RSD”:

(1) $$Composite\;RSD=\sqrt{{\left(Weight\;RSD\right)}^{2}+{\left(Potency\;RSD\right)}^{2}}$$

where “Weight RSD” is the relative standard deviation of bulk mini-tablet weights, and “Potency RSD” is the relative standard deviation of bulk mini-tablet potency (normalized by weight). Note the composite RSD is, therefore, a property of bulk mini-tablets rather than sachets. This simplification is supported mathematically (see Supplementary Materials, Section S1.4) and qualitatively (Figure 3) by the observation that potency and weight RSDs contribute equally to CU failure probability. Reduction of weight RSD and potency RSD to a single composite RSD facilitated simulation/visualization of fill count and fill error probability as contour plots. These were modeled with ranges of 0–15% for composite RSD, 1 to 10 for fill count, and 0% to 10% fill error probability. 

### 2.4. Contour Plot Visualizations

**Figure 4:**
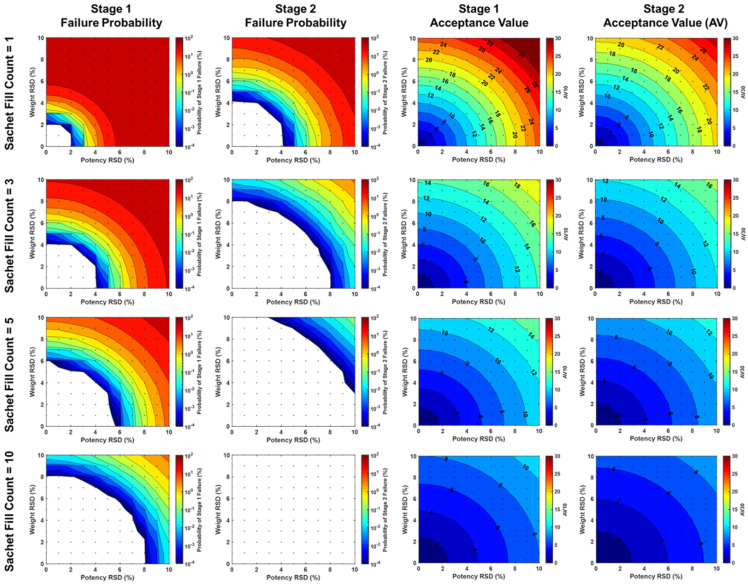
Probability of stage 1 and stage 2 content uniformity batch failure and acceptance values for 0% fill error probability represented as a function of modeled bulk mini-tablet weight RSD (vertical axes), bulk mini-tablet potency RSD (horizontal axes), and sachet fill count (rows). Tabulated values may be found in Supplementary Materials Section S2.1.

**Figure 5:**
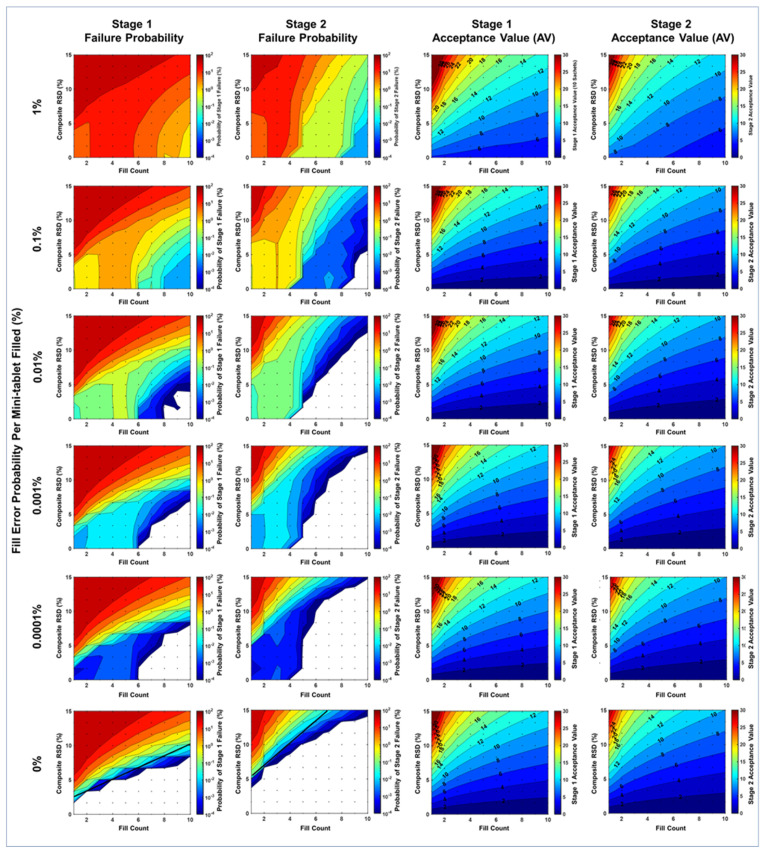
The probability of stage 1 and stage 2 content uniformity batch failure and acceptance values (AV) modeled as a function of sachet fill count (horizontal axes), composite RSD of bulk mini-tablets (vertical axes), and fill error probability per mini-tablet filled (rows). Tabulated values may be found in Supplementary Materials Section S2.2.

**Figure 6:**
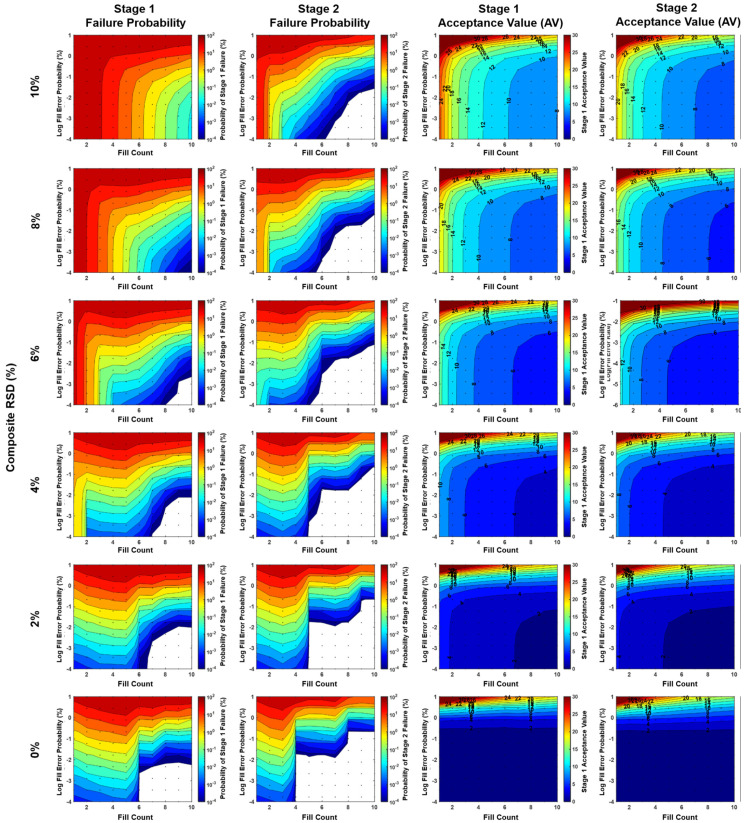
The probability of stage 1 and stage 2 content uniformity batch failure and acceptance values (AV) are modeled as a function of sachet fill count (horizontal axes), fill error probability per mini-tablet filled (vertical axes), and composite RSD (rows). Tabulated values may be found in Supplementary Materials Section S2.3.

In contour plots shown in Figure 3, Figure 4, Figure 5 and Figure 6, the probability of CU failure or acceptance values (colors on contour plots) are logarithmically and linearly spaced, respectively. Composite RSD refers to the content of bulk mini-tablets rather than to the content of sachets. Fill error probability is the probability of a single error occurring for each mini-tablet filled into a sachet (i.e., it is not the probability that a sachet would have an error). Grid points represent the points at which model calculations were performed. White space indicates values below the model calculation threshold of 0.0001% (1/1,000,000).

## 3. Results

The probability of failing CU testing was modeled as a function of weight RSD, potency RSD, fill error probability, and fill count. Weight and potency RSD impacted CU outcomes similarly and proportionally to the root sum of their squares (Figure 3 and Figure 4). Fill count and fill error probability was found to impact CU over a range of composite RSDs (Figure 5 and Figure 6). Tabulated values for Figure 4, Figure 5 and Figure 6 may be found in Supplementary Materials Section S2.

Parameter ranges modeled included 1–10 fill count, 0–10% weight RSD and potency RSD (0–15% composite RSD), and 0–10% fill error probability per mini-tablet. Over these ranges, the probability of stage 1 and stage 2 CU failure was observed from <1 × 10^−4^% to nearly 97.6% (Figure 5). However, for fill counts of at least five mini-tablets per sachet, the maximum observed failure probability was lower at 15.9%. 

Consider the effect of fill count on a representative product having 5% weight RSD and potency RSD and 0.1% fill error probability per mini-tablet. The maximum CU failure probability for this product was 8.23% for fill counts of 1–4 but was 0.283% for fill counts of 5–10. For a well-controlled product with 2% weight RSD and potency RSD and <0.1% fill error probability per mini-tablet, the maximum CU failure probability was 0.497% for fill counts of 1–4 but was 0.00435% fill counts of 5–10. 

The effect of fill error probability is likewise pronounced. For the same representative product above having a greater fill error probability per mini-tablet of 1% rather than 0.1%, the CU failure probability is 22.2% for fill counts 1–4, and 3.96% for fill counts 5–10. Assuming a smaller fill error probability per mini-tablet of 0.01%, the CU failure probability is 6.64% for fill counts 1–4, and 0.0264% for fill counts 5–10.

### 3.1. Effect of Weight RSD, Potency RSD, and Fill Count on CU in the Absence of Fill Errors

In the absence of fill errors, increasing potency RSD and weight RSD adversely impacted CU stage 1 and stage 2 failure probabilities and AV values ranging from 1% to 10% (Figure 4). At a 10-count target fill, the probability of stage 2 failure was below the model threshold of detection (<0.0001%) for all combinations of potency RSD and weight RSD simulated (0 through 10%). Stage 1 failure probability is always greater than stage 2 failure probability, as the former always precedes the latter.

### 3.2. CU Failure Probability and AV as a Function of Fill Count, Composite RSD, and Fill Error Probability

CU failure probability was sensitive to changes in fill count, composite RSD, and fill error probability (Figure 5). Generally, increases in fill count resulted in a lower probability of CU failure, while increases in composite RSD and fill error probability resulted in an increased probability of failure. Most notably, a significant decrease in stage 2 failure probability (>100-fold) was found at the transitions between n = 4 and n = 5 count and n = 8 to n = 9 counts for low composite RSDs (<5%). This phenomenon is an artifact resulting from the application of the USP <905> specification’s anticipation of Gaussian content variance to a drug product with non-Gaussian variance arising from discrete fill errors. Interestingly, increasing the fill count sometimes adversely impacted CU failure probability. For example, in the cases of 0.0001% to 1% fill error probabilities and low composite RSDs (0–5%), increasing fill count from n = 1 to n = 3 or (and occasionally from n = 5 to n = 7) increased CU failure probability slightly. See Supplementary Materials Section S2 for tabulated values. 

CU failure probability plots appear to have two distinct regions: (1) a region of high failure probabilities corresponding with large composite RSD values, and (2) a region of lower failure probabilities sensitive to fill errors, corresponding with lower composite RSD values. Plots for 0% fill error probability provide an important control illustrating this demarcation (increasing concave downward contours). The low composite RSD region changes with fill error probability, while the high composite RSD region does not. This suggests that composite RSD dominates CU failure predictably, but in a manner dependent on fill count.

While CU failure probability was sensitive to all parameters modeled, AV values varied little across fill error probabilities. The similarity in AV values across fill error probabilities from 0% to 0.1% suggests that fill errors in this range would not likely be reflected in AV values experimentally, even if large sample sizes were used.

## 4. Discussion

### 4.1. Model Impact on Pharmaceutical Development

Mini-tablets in sachets hold promise as a flexible dosage form amenable to a wide range of doses and pediatric populations. They are especially relevant considering ongoing efforts to improve pediatric health, including the passage and amendment of the Pediatric Research Equity Act (PREA) encouraging pediatric product development [18,19]. In this study USP <905> CU pass/fail conditions have been modeled as a function of selected theoretical process and fill count parameters. These data may, for example, be useful for (1) risk quantification in research and development, (2) making a data-driven decision for fill count selection in early product development, (3) determining formulation specifications and control limits for weight and potency variance, and (4) determining maximum acceptable fill error probability for equipment qualification or process control limits.

### 4.2. Model Assumptions and Limitations

Certain model assumptions must be considered when translating the present study findings to product development. First, weight and potency were assumed to be normally distributed, though product-specific log-normal or uniform distributions may be preferred. Additionally, fill error probability was modeled with a Binomial distribution which is appropriate for discrete miscount filling errors involving whole mini-tablets. However, broken or chipped mini-tablets are possible during sachet filling. In such circumstances, a distribution for broken mini-tablet size would be warranted. Related to this, the probability of over and under-fill errors was assumed to be equal, though they often occur by different mechanisms. As a result, they may occur with unequal probabilities of over and under-filling. Even the assumption that fills errors occur independently of one another is imperfect as transient mechanical defects in machinery could lead to grouping of fill errors over time. Finally, fill errors may not be independent of bulk mini-tablet weight and potency as was assumed, especially in cases where large deviations may interfere with filling machinery. In light of these considerations, the assumptions are reasonable for an average mini-tablet product where discrimination of orders of magnitude failure probabilities is desired.

### 4.3. Explanation for Unintuitive Impact of Fill Count on CU Failure

**Figure 7:**
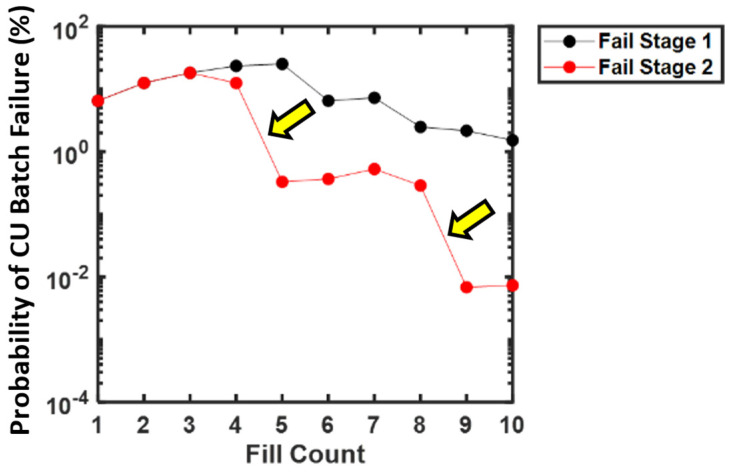
Probability of stage 1 or stage 2 CU failure for an example product having 2% composite RSD and 1% fill error probability, illustrating sharp probability transitions (arrows) occurring between sachet fill counts of 4 to 5 mini-tablets and 8 to 9 mini-tablets. Note these data are the same as in Figure 5 and Figure 6 but represented in two dimensions for ease of visualization.

Greater sachet fill counts generally lead to lower predicted probabilities of CU failure. Two unintuitive observations are worth noting. First, CU failure probability decreases sharply when transitioning from 4 to 5 counts and from 8 to 9 counts at low root square RSDs (Figure 7). Second, increasing the fill count may sometimes worsen CU failure probability (Figure 5). Note the increase in failure probability at a fill count of 2–4 compared to a fill count of 1 while holding composite RSD constant between 0% and 5% for fill error probabilities > 0.001%.

To the first point, a marked transition existed at low weight RSD and potency RSD where the probability of CU failure is as much as 10-fold lower for a 5-count compared to a 4-count fill. This sharp transition is an artifact of non-Gaussian content variance due to filling count errors. Typically, the variance of sachet contents should decrease proportionally to the reciprocal of fill count, as sachet content is the sum of individual mini-tablet contents (normal random variables). However, due to filling count errors: (1) CU failure is driven by miscounts rather than weight or potency variation at low standard deviations, and (2) failure occurs when one or more unit label claim is outside of ±25% of the mean. A single miscount for a target 4-count fill results in a ±25% dosage error on average (triggering batch failure according to USP <905> criteria), while for a 5-count, it would result in a ±20% dosage error, which is below the critical USP <905> threshold. A careful observation of high fill error probability shows a similar transition between 8-count and 9-count fill targets for the same reasons. An 8-count product may fail when two miscounts occur (2 in 8, also ±25%). However, two miscounts would not cause the failure of a 9-count product (2 in 9, 22%). The probability of observing two miscounts is much lower than for one miscount and only appreciably occurs at large fill error probabilities.

To the second point, CU failure probability occasionally increases with increasing fill count, as in the case of moving from 1-count to 4-count. This is observed for non-zero fill error probability and low composite RSD (Figure 5). One might have contrarily expected that greater fill counts result in lower CU failure probability as assay variance should decrease. However, fill errors (rather than product weight or potency variance) drive failure in the low standard deviation regime. Consider fill counts ranging from 1 to 4. A single fill error will cause batch failure in CU testing for any of these, as the assay would deviate by more than ±25%. The probability of observing just one fill error is proportional to the number of chances for a fill error to occur. Since each mini-tablet independently carries a miscount probability, larger fill counts carry an increased risk of observing a single error. Roughly, the probability of a single miscount for a 4-count fill is four times that of a 1-count fill, thus explaining the unintuitive increase in CU failure probability on that range.

### 4.4. AV Values Alone Are Imperfect Indicators of CU Failure Probability

Mean AV values were nearly identical for all fill error probabilities modeled (Figure 6), despite marked differences in CU failure probabilities across these fill error probabilities. Therefore, AV values are not, on average, sensitive to fill errors and are poor indicators of product CU risk. It should be cautioned against using AV values alone for risk assessments or comparison of product dosage uniformity. To illustrate this point, it is noted that a 4-count product with 5% composite RSD will, on average, have the same AV value (AV = 6%) across all fill error probabilities modeled, yet the probability of failing CU across these error probabilities range from <0.0001% at 0% fill error probability to 1% at 0.1% fill error probability. Failure to consider the insensitivity of AV values to fill errors may result in false confidence in product risk analyses. This insensitivity may be explained by the fact that mean AV values are weakly affected by low-frequency failure events. Though occurring at low enough frequency as to be unresolved by AV values, a 1% CU failure probability, for example, is meaningful to product developers.

### 4.5. Model Use Cases Illustrated as Scenarios Encountered in Product Development

Use cases are shown to illustrate how model results may inform product development. Note additional use cases are included in Supplementary Materials. Generally, these examples are concerned with stage 2 failure probabilities, though the same principles apply to stage 1 failure probabilities—the appropriate plot simply needs to be used from Figure 5 and Figure 6. We advise caution when interpreting mean AV values, as they were found to be insensitive to fill error probability in some cases. They may be used to predict the expected value, which would be observed experimentally but should not be used to predict fill error probabilities.

#### 4.5.1. Use Case 1

A scientist is developing a new mini-tablet product and wishes to know the probability of failing CU. Determine the probability of failing CU based on the following measured outcomes from product development for a product intended to be dosed with four mini-tablets per sachet:

- Weight RSD = 5.0%
- Potency RSD = 5.0%
- Fill error probability = 0.001%
- Fill count = 4
- Probability of failing CU = ?

Solution:

Step 1. Calculate composite RSD:

$$Composite\;RSD=\sqrt{{\left(Weight\;RSD\right)}^{2}+{\left(Potency\;RSD\right)}^{2}}$$

$$Composite\;RSD=\sqrt{5.0{\%}^{2}+5.0{\%}^{2}}=7.1\%$$

Step 2. Use model results (Figure 5) and calculated composite RSD above (7.1%) to determine the probability of stage 2 failure.


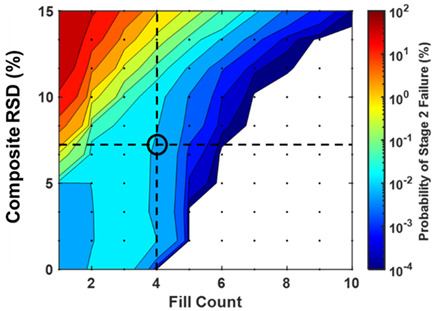


Solution: The probability of failing CU is approximately 0.01% for the specified product configuration.

#### 4.5.2. Use Case 2

A scientist is developing a new mini-tablet product, and the weight RSD and potency RSD of the process were measured experimentally. They wish to calculate the fill counts for which the probability of failing CU is less than 0.01% given the known parameters:

- Weight RSD = 2.0%
- Potency RSD = 1.0%
- Fill error probability = 0.01%
- Fill count = ?
- Probability of failing CU = <0.01%

Step 1: Calculate the composite RSD:

$$Composite\;RSD=\sqrt{{\left(Weight\;RSD\right)}^{2}+{\left(Potency\;RSD\right)}^{2}}$$

$$Composite\;RSD=\sqrt{1.0{\%}^{2}+2.0{\%}^{2}}=2.2\%$$

Step 2: Determine the fill counts for which the probability of failing CU is less than 0.01% at 2.2% composite RSD using Figure 5: 


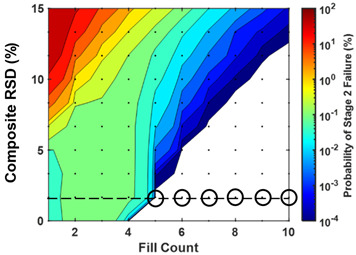


Conclusions: The fill counts for which the probability of failure would be less than 0.01% for the given process parameters are 5, 6, 7, 8, 9, and 10 counts (and likely >10 counts as well).

### 4.6. Future Applications

Future studies may extend to more complex scenarios, such as combination products comprised of mini-tablets containing different drug substances. For example, a product with four mini-tablets containing one drug substance and four mini-tablets containing another would exhibit greater failure risk as the probability of success is the product of the probabilities of success for each of the two types individually (assuming independence of events).

Additionally, this study considered mini-tablets distributed with batch mean weight and potency centered at the intended targets, but deviations in batch mean values frequently occur (even if by small amounts for well-controlled processes). Future modeling may determine the impact of these deviations on CU towards setting process control limits.

## 5. Conclusions

CU failure probability was modeled as a function of potency RSD, weight RSD, fill count, and fill error probability. Generally, fill counts of fewer than five mini-tablets per sachet should be avoided.

The results may inform product development decisions, such as the appropriate selection of fill counts to manage CU failure probability risk. Additionally, the results may be consulted to prioritize risk reduction strategies during formulation and process development (i.e., directing effort toward minimizing potency RSD, weight RSD, or fill error probability). Beyond informing drug product development, the model adds to the existing body of statistical modeling literature a method for CU prediction which can be adapted in future studies to evolving regulatory requirements or process limitations/advances.

## Data Availability

Data is contained within the article or Supplementary Materials.

## Supplementary Materials

The following supporting information can be downloaded at: https://www.mdpi.com/article/10.3390/pharmaceutics15020594/s1, Figure S1: Visualization of potency and weight variances used for modeling. Each point represents a single simulated mini-tablet plotted with respective weight and potency value (note, data for illustration only, not simulated). The weight variance is measured from the distribution of weights. The potency variance is the variance of the residuals of the regression of potency against weight (i.e., the weight normalized potency); Table S1: Conditions for determining USP <905> parameter, M, used in the calculation of acceptance value (AV). References [20,21] have been cited in the Supplementary Materials.
