# Supplementary material for: Pediatric Mini-Tablets: Predicting the Hidden Risk of Fill Errors
Source: Pharmaceutics. 2023 Feb 10;15(2):594. doi: 10.3390/pharmaceutics15020594 (PMC9961976; doi:10.3390/pharmaceutics15020594)
Supplement: Supplementary file 1 [file pharmaceutics-15-00594-s001.zip › pharmaceutics-2075419-supplementary.pdf]

Pediatric Mini-tablets: Predicting the Hidden Risk of Fill Errors

Brandon G. Gerberich *, Grace A. Okoh, James C. DiNunzio and Michael B. Lowinger

Oral Formulation Sciences, Merck & Co., Inc., Kenilworth, NJ 07065, USA

S1. Methods

S1.1 Calculation of Normalized Sachet Content

Normalized sachet content (χ) was modeled as the product of normally distributed random individual mini-tablet weights (w), and potency values (A) normalized by the target weight (w_{Target}) and assay (A_{Target}) values:

$$w = \frac{N(\mu_w, \sigma_w^2)}{w_{Target}} \tag{S1}$$

$$A = \frac{N(\mu_A, \sigma_A^2)}{A_{Target}} \tag{S2}$$

$$\chi = w \times A \tag{S3}$$

where the notation N(x, y) indicates a random normal variable with mean x and variance y, μ_w is the observed mean weight of individual mini-tablets, σ_w^2 is the observed variance of individual mini-tablets, μ_A is the observed mean assay of individual mini-tablets, and σ^2_A is the observed assay variance of individual mini-tablets. In this study, we represent variance as relative standard deviation (RSD), as is common practice in the field, which is simply the standard deviation divided by the mean:

$$Weight\ RSD = \frac{\sigma_w}{\mu_w} \tag{S4}$$

$$Potency\ RSD = \frac{\sigma_A}{\mu_A} \tag{S5}$$

Experimentally, sachet content and potency are often measured by liquid chromatography techniques to calculate content uniformity. It is worth noting that additional variance theoretically results from experimental measurement but was not accounted for in this model.

S1.2. Normalization of Individual Mini-tablet Weight and Weight-correction of Potency

Individual granule weights were normalized by the intended target weight. For example, a mini-tablet weighing 11 mg with intended target 10 mg weight would have a normalized weight of 1.1 (110%). Therefore, the model results do not change for various target weights or potencies.

Confidential

Furthermore, individual mini-tablet potencies were weight-normalized so that the normalized potency may be interpreted as blend uniformity, or in other words, the superpotency or subpotency of the individual mini-tablet irrespective of its weight. To help illustrate this point, Figure S1 shows graphically the weight and potency variances. Readers intending to use the model results to predict CU outcomes should record experimentally the weight RSD for a sample of mini-tablets and calculate the potency RSD as a weight-normalized value.

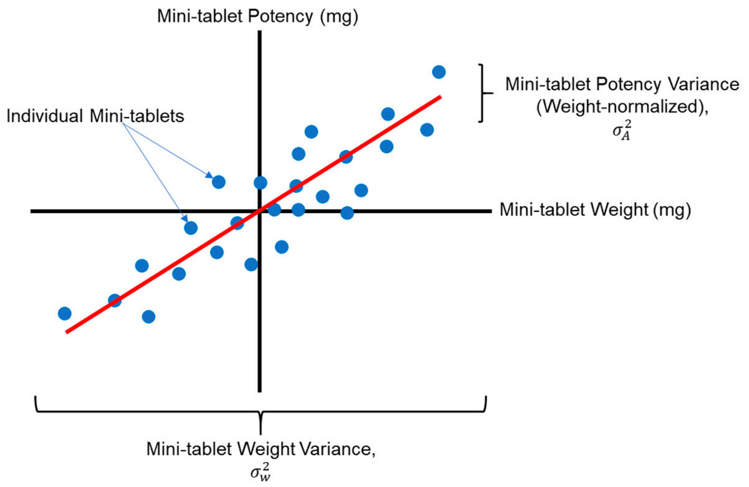

Figure S1: Visualization of potency and weight variances used for modeling. Each point represents a single simulated mini-tablet plotted with respective weight and potency value (note, data for illustration only, not simulated). The weight variance is measured from the distribution of weights. The potency variance is the variance of the residuals of the regression of potency against weight (i.e., the weight normalized potency).

S1.3. Fill Errors Modeled Using Binomial Distribution

Weight and potency are continuous variables and assumed to be normally distributed. In contrast, fill count errors were assumed to be discrete and thus Binomial distributed (1). Probability, $P_{k\ miscounts}$, of encountering 'k' miscounts in 'n' filled mini-tablets was calculated using the Binomial probability:

$$P_{k\ miscounts} = {}_nC_k \times (1-p)^{(n-k)} \times p^k \qquad \text{S6}$$

where p is the fill error probability represented as the probability of a miscount per mini-tablet filled into a sachet. Implicitly, this distribution assumes independence of miscount events and accounts for the possibility of multiple miscounts per sachet.

The following example illustrates implementation of Equation S6 in the model. Assume a product is manufactured with target count of 3 mini-tablets per sachet. Assume the probability of a miscount for each individual mini-tablet being filled is 1 in 100 (1%)

- n = 3
- p = 0.01

- k is any number from 0 to 3
- $P_{k\ miscounts}$ is the probability of observing a fill count of 'k'

By iterating through values of k from 0 to 3, the probability of observing k miscounts is calculated using Equation S6:

$$P_{0\ miscounts} = {}_3C_0 \times (1-0.01)^3 \times 0.01^0 = 0.970299 \quad \text{S6.1}$$

$$P_{1\ miscounts} = {}_3C_1 \times (1-0.01)^2 \times 0.01^1 = 0.029403 \quad \text{S6.2}$$

$$P_{2\ miscounts} = {}_3C_2 \times (1-0.01)^1 \times 0.01^2 = 0.000297 \quad \text{S6.3}$$

$$P_{3\ miscounts} = {}_3C_3 \times (1-0.01)^0 \times 0.01^3 = 0.000001 \quad \text{S6.4}$$

1) The above probabilities were used to randomly generate the number of miscounts (in this case, a number 0 to 3) assuming the errors are all in the same direction. The above equation indicates the number of errors in a sachet but not the "direction" of the errors (i.e. overfill versus underfill). It is important to account for the net effect of errors in both directions (the difference between the overfills and underfills) because they may cancel each other out, resulting in a sachet fill count error which is less than the total number of fill errors.

2) To account for the possibility that the fill count errors are not in the same direction, the algorithm then assigns a +1 or -1 value randomly with 50% probability for each error indicating an overfill (+1) or underfill (-1). The sum of under and over-fills is calculated and added to the target to arrive at the randomly generated sachet fill count. For example, if 3 errors were randomly generated using Equation S6, and these were assigned values of +1, -1, and +1, respectively, then the sachet would be assigned a fill count of 4 since for 3 fill events, two resulted in an extra mini-tablet and one resulted in a missing mini-tablet.

Mean of individual contents, $\overline{X}$, was calculated as the arithmetic mean of individual sachet contents, 'χ' for each set of sachets, and an acceptance value was calculated according to USP <905> specifications:

$$AV = \left|M - \overline{X}\right| + ks \quad \text{S7}$$

where AV is the acceptance value, 'M' is a reference value (Table S1, with criteria reproduced from USP <905>), 'k' is the acceptability constant, and 's' is the sample standard deviation of individual contents. The acceptance value was used to determine batch passage or failure according to USP <905> criteria as follows and outlined diagrammatically in Figure S1.

C Confidential

Table S1: Conditions for determining USP <905> parameter, M, used in the calculation of acceptance value (AV).

	Conditions	Value
If target content is ≤ 101.5%, i.e., if batch target content is equal to the target value of 100% (as was assumed in this study)	If 98.5% ≤ $\overline{X}$ ≤ 101.5%, then	$M = \overline{X}$ (AV = ks)
	If $\overline{X}$ ≤ 98.5%, then	$M = 98.5\%$ (AV = 98.5 - $\overline{X}$ + ks)
	If $\overline{X}$ ≥ 101.5%, then	$M = 101.5\%$ (AV = $\overline{X}$ - 101.5 + ks)

Stage 1 passing criteria:

$$AV < L1 \qquad \text{S8}$$

where L1 equals 15%. Stage 2 passing criteria:

$$AV < L2 \qquad \text{S9}$$

where L1 = 25%. To pass Stage 2, all individual contents must also meet the following criteria:

$$\chi > (1 - (0.01)(L2))M \qquad \text{S10}$$

$$\chi < (1 + (0.01)(L2))M \qquad \text{S11}$$

Finally, the probability of failing either stage 1 or stage 2 was calculated as the number of failed batches divided by the number of simulated batches:

$$p_{failure} = \frac{\# \, failed \; batches}{\# \, simulations} \times 100\% \qquad \text{S12}$$

The model simulated trials successively until the 95% confidence intervals of failure probability were within 2-fold multiples of the mean. For example, if after 1,000,000 simulations, the failure probability was calculated to be 5x10^{-3}% with 95% confidence intervals of 2x10^{-3}% and 12x10^{-3}%, the model would continue increasing the number of iterations until the 95% confidence intervals fell within 2.5 x 10^{-3}% and 10 x 10^{-5}%. Failure probabilities with upper 95% confidence interval converging below 1x10^{-4}% were recorded as below limit of detection and appear as white space on the figure countour maps.

S1.4. Simplification of Weight and Potency RSD Parameters

In this study, assay was calculated as the product of normally distributed mini-tablet weight and normally distributed mini-tablet potency. Weight and potency were each normalized by target weight and potency, respectively, so that the expected value of the means was equal to 1. Here the simplification of weight RSD and potency RSD into a single parameter, composite RSD, is justified mathematically. The composite RSD is justified mathematically since it is a simplification of the product of two random normal variables (as is present in this study). As defined in the main text, composite RSD is:

C Confidential

$$Composite\ RSD = \sqrt{(Weight\ RSD)^2 + (Potency\ RSD)^2} \quad \text{S13}$$

The notation may be generalized for any two random normal variables having means μ_w and μ_A and variances σ_w^2 and σ_p^2, respectively as in Section S1.1. The composite mean and variance will be defined as μ_C and σ_C^2:

$$\frac{\sigma_C}{\mu_C} = \sqrt{\left(\frac{\sigma_W}{\mu_W}\right)^2 + \left(\frac{\sigma_A}{\mu_A}\right)^2} \quad \text{S14}$$

In this study, mean mini-tablet weight and potency were equal to 1 since they were normalized by target weight and potency. Therefore, the relative standard deviations (defined as standard deviation divided by mean) are equivalent to the standard deviations:

$$\sigma_C = \sqrt{\sigma_W^2 + \sigma_A^2} \quad \text{S15}$$

It has been shown previously that the variance of the product of two random normal variables is as follows (2):

$$\sigma^2 = \mu_1^2\sigma_1^2 + \mu_2^2\sigma_2^2 + \sigma_1^2\sigma_2^2 \quad \text{S16}$$

Where σ^2 is the variance of the product of random normal variables, μ_1 and μ_2 are the means of the first and second random normal variables, respectively, and σ_1^2 and σ_2^2 are the variances of the first and second random normal variables respectively. Since means were equal to 1 in the present study, equation S16 simplifies to:

$$\sigma^2 = \sigma_1^2 + \sigma_2^2 + \sigma_1^2\sigma_2^2 \quad \text{S17}$$

If the standard deviations of each random normal variable are small (for example ≤0.15 as in this study), then their product, $\sigma_1^2\sigma_2^2$, is much smaller than their sum, $\sigma_1^2 + \sigma_2^2$:

$$\sigma_1^2\sigma_2^2 = (0.15)^2(0.15)^2 = 0.000501625 \quad \text{S18}$$

$$\sigma_1^2 + \sigma_2^2 = (0.15)^2 + (0.15)^2 = 0.045 \quad \text{S19}$$

The product may be assumed negligible compared to the sum in this study, and therefore the approximation for variance of the product of two random normal variables in Equation S17 further simplifies to:

$$\sigma^2 = \sigma_1^2 + \sigma_2^2 \quad \text{S20}$$

Final rearrangement to calculate standard deviation yields:

$$\sigma = \sqrt{\sigma_1^2 + \sigma_2^2} \quad \text{S21}$$

C Confidential

Which is identical to Equation S15 and therefore identical to the composite RSD. Thus, it is shown that for the values of mean and variance used in this study, the composite RSD is a reasonable approximation for the RSD of the product of two random normal variables.

S2. Results

The tables below contain values calculated by the model and used to generate the contour maps in the main text.

S2.1. Tables of calculated values corresponding to Figure 4 in main manuscript

Stage 1 Failure Probability (%)
Fill Count = 1

Composite RSD (%) \ Fill Count	1	2	3	4	5	6	7	8	9	10
**15.00**	96.6	96.7	97.1	97.7	98.2	98.8	99.2	99.4	99.7	99.8
**13.33**	92.4	92.7	93.8	95.1	96.4	97.6	98.4	99.1	99.4	99.6
**11.67**	83.2	84.1	86.5	89.6	92.8	95.3	97.2	98.3	99.0	99.5
**10.00**	64.2	66.3	71.6	78.8	85.7	91.2	94.9	97.1	98.5	99.2
**8.33**	33.8	37.1	46.5	59.7	73.3	84.1	91.2	95.3	97.6	98.8
**6.67**	6.63	8.89	17.3	33.8	54.9	73.3	85.8	92.8	96.5	98.2
**5.00**	0.0772	0.229	1.94	11.5	33.8	59.7	78.7	89.7	95.1	97.7
**3.33**	<1.00E-04	<1.00E-04	0.0199	1.95	17.4	46.5	71.7	86.4	93.8	97.2
**1.67**	<1.00E-04	<1.00E-04	<1.00E-04	0.223	8.89	37.1	66.2	84.1	92.8	96.8
**0.00**	<1.00E-04	<1.00E-04	<1.00E-04	0.0752	6.61	33.7	64.2	83.0	92.4	96.6

Stage 2 Failure Probability (%)
Fill Count = 1

Composite RSD (%) \ Fill Count	1	2	3	4	5	6	7	8	9	10
**15.00**	29.5	30.8	34.5	40.8	49.6	60.0	71.0	80.9	88.6	93.9
**13.33**	12.0	12.9	15.6	20.8	28.7	39.8	53.3	67.1	79.6	88.6
**11.67**	3.03	3.42	4.82	7.79	13.2	22.1	34.9	50.9	67.2	80.8
**10.00**	0.351	0.425	0.830	1.91	4.58	10.1	20.0	34.9	53.3	70.9
**8.33**	0.00900	0.0140	0.0558	0.255	1.09	3.73	10.1	22.1	39.9	60.0
**6.67**	<1.00E-04	<1.00E-04	0.000746	0.0130	0.167	1.09	4.54	13.3	28.9	49.6
**5.00**	<1.00E-04	<1.00E-04	<1.00E-04	0.000288	0.0155	0.264	1.92	7.83	20.8	40.8
**3.33**	<1.00E-04	<1.00E-04	<1.00E-04	<1.00E-04	0.000794	0.0527	0.818	4.84	15.7	34.7
**1.67**	<1.00E-04	<1.00E-04	<1.00E-04	<1.00E-04	<1.00E-04	0.0153	0.430	3.45	12.9	31.0
**0.00**	<1.00E-04	<1.00E-04	<1.00E-04	<1.00E-04	<1.00E-04	0.00950	0.343	3.04	12.0	29.5

C Confidential

Stage 1 Acceptance Value (%)
Fill Count = 1

Fill Count

Composite RSD (%)	1	2	3	4	5	6	7	8	9	10
**15.00**	25	25	26	26	27	29	30	32	34	36
**13.33**	22	22	23	24	25	26	28	30	32	34
**11.67**	19	20	20	21	22	24	26	27	30	32
**10.00**	17	17	17	18	20	22	23	26	28	30
**8.33**	14	14	15	16	18	20	22	24	26	29
**6.67**	11	11	12	14	16	18	20	22	25	27
**5.00**	8.4	8.8	10	12	14	16	18	21	24	26
**3.33**	5.7	6.3	7.9	10	12	15	17	20	23	26
**1.67**	2.9	4.1	6.3	8.8	11	14	17	20	22	25
**0.00**	0	2.9	5.7	8.4	11	14	17	19	22	25

Stage 2 Acceptance Value (%)
Fill Count = 1

Fill Count

Composite RSD (%)	1	2	3	4	5	6	7	8	9	10
**15.00**	21	21	21	22	23	24	25	26	28	29
**13.33**	18	19	19	20	21	22	23	24	26	28
**11.67**	16	16	17	18	19	20	21	23	24	26
**10.00**	14	14	15	15	17	18	20	21	23	25
**8.33**	12	12	12	13	15	16	18	20	22	24
**6.67**	9.4	9.6	10	12	13	15	17	19	21	23
**5.00**	7.1	7.5	8.5	9.9	12	13	15	18	20	22
**3.33**	4.7	5.3	6.7	8.5	10	12	15	17	19	21
**1.67**	2.4	3.3	5.3	7.5	9.6	12	14	16	19	21
**0.00**	0	2.4	4.7	7.1	9.4	12	14	16	18	21

Stage 1 Failure Probability (%)
Fill Count = 3

Fill Count

Composite RSD (%)	1	2	3	4	5	6	7	8	9	10
**15.00**	40.3	41.3	44.4	49.5	55.8	62.8	70.0	76.8	82.6	87.3
**13.33**	21.5	22.7	26.1	31.7	39.3	48.5	58.3	67.6	75.9	82.6
**11.67**	7.34	8.12	10.6	15.4	22.8	32.8	44.7	56.6	67.6	76.8
**10.00**	1.08	1.33	2.30	4.73	9.79	18.5	30.6	44.6	58.3	70.1
**8.33**	0.0330	0.0472	0.162	0.693	2.73	8.16	18.5	32.8	48.5	62.9
**6.67**	<1.00E-04	<1.00E-04	0.00132	0.0314	0.418	2.73	9.78	22.8	39.3	55.8
**5.00**	<1.00E-04	<1.00E-04	<1.00E-04	0.000125	0.0321	0.693	4.71	15.3	31.6	49.4
**3.33**	<1.00E-04	<1.00E-04	<1.00E-04	<1.00E-04	0.00145	0.162	2.30	10.6	26.1	44.5
**1.67**	<1.00E-04	<1.00E-04	<1.00E-04	<1.00E-04	<1.00E-04	0.0509	1.33	8.09	22.7	41.4
**0.00**	<1.00E-04	<1.00E-04	<1.00E-04	<1.00E-04	<1.00E-04	0.0302	1.10	7.31	21.6	40.4

C Confidential

Stage 2 Failure Probability (%)
Fill Count = 3

Fill Count

Composite RSD (%)	1	2	3	4	5	6	7	8	9	10
**15.00**	0.0240	0.0277	0.0405	0.0725	0.157	0.341	0.756	1.59	3.15	5.72
**13.33**	0.00106	0.00158	0.00326	0.00791	0.0246	0.0766	0.231	0.603	1.46	3.13
**11.67**	<1.00E-04	<1.00E-04	0.000114	0.000597	0.00287	0.0129	0.0510	0.198	0.603	1.62
**10.00**	<1.00E-04	<1.00E-04	<1.00E-04	<1.00E-04	0.0002	0.00113	0.0106	0.0572	0.223	0.764
**8.33**	<1.00E-04	<1.00E-04	<1.00E-04	<1.00E-04	<1.00E-04	<1.00E-04	0.00138	0.0120	0.0787	0.346
**6.67**	<1.00E-04	<1.00E-04	<1.00E-04	<1.00E-04	<1.00E-04	<1.00E-04	0.000160	0.00247	0.0254	0.156
**5.00**	<1.00E-04	<1.00E-04	<1.00E-04	<1.00E-04	<1.00E-04	<1.00E-04	<1.00E-04	0.000432	0.00841	0.0698
**3.33**	<1.00E-04	<1.00E-04	<1.00E-04	<1.00E-04	<1.00E-04	<1.00E-04	<1.00E-04	0.000150	0.00257	0.0397
**1.67**	<1.00E-04	<1.00E-04	<1.00E-04	<1.00E-04	<1.00E-04	<1.00E-04	<1.00E-04	<1.00E-04	0.00208	0.0254
**0.00**	<1.00E-04	<1.00E-04	<1.00E-04	<1.00E-04	<1.00E-04	<1.00E-04	<1.00E-04	<1.00E-04	0.00158	0.0217

Stage 1 Acceptance Value (%)
Fill Count = 3

Fill Count

Composite RSD (%)	1	2	3	4	5	6	7	8	9	10
**15.00**	14	14	15	15	16	16	17	18	19	20
**13.33**	13	13	13	14	14	15	16	17	18	19
**11.67**	11	11	12	12	13	14	15	16	17	18
**10.00**	9.6	9.8	10	11	12	12	14	15	16	17
**8.33**	8.1	8.2	8.7	9.4	10	11	12	14	15	16
**6.67**	6.5	6.7	7.3	8.1	9.1	10	12	13	14	16
**5.00**	5.0	5.2	5.9	6.9	8.1	9.4	11	12	14	15
**3.33**	3.3	3.7	4.7	5.9	7.3	8.7	10	12	13	15
**1.67**	1.7	2.3	3.7	5.2	6.7	8.2	9.8	11	13	14
**0.00**	0	1.7	3.3	5.0	6.5	8.1	9.6	11	13	14

Stage 2 Acceptance Value (%)
Fill Count = 3

Fill Count

Composite RSD (%)	1	2	3	4	5	6	7	8	9	10
**15.00**	12	12	12	13	13	14	14	15	16	17
**13.33**	11	11	11	11	12	13	13	14	15	16
**11.67**	9.5	9.6	9.8	10	11	12	12	13	14	15
**10.00**	8.1	8.3	8.6	9.1	9.7	11	11	12	13	14
**8.33**	6.8	6.9	7.3	7.9	8.7	9.6	11	12	13	14
**6.67**	5.5	5.6	6.1	6.8	7.7	8.7	9.7	11	12	13
**5.00**	4.1	4.3	4.9	5.8	6.8	7.9	9.1	10	11	13
**3.33**	2.7	3.1	3.9	4.9	6.1	7.3	8.6	9.8	11	12
**1.67**	1.4	1.9	3.1	4.3	5.6	6.9	8.3	9.6	11	12
**0.00**	0	1.4	2.7	4.1	5.5	6.8	8.1	9.5	11	12

C Confidential

Stage 1 Failure Probability (%)
Fill Count = 5

Fill Count

Composite RSD (%)	1	2	3	4	5	6	7	8	9	10
**15.00**	7.04	7.48	8.90	11.5	15.6	21.3	28.8	37.4	46.8	56.1
**13.33**	1.73	1.93	2.62	4.05	6.66	11.0	17.5	26.0	36.1	46.8
**11.67**	0.187	0.225	0.390	0.849	1.96	4.42	9.00	16.3	26.0	37.4
**10.00**	0.00444	0.00628	0.0190	0.0751	0.333	1.25	3.77	9.00	17.5	28.7
**8.33**	<1.00E-04	<1.00E-04	<1.00E-04	0.00252	0.0270	0.233	1.26	4.39	11.0	21.3
**6.67**	<1.00E-04	<1.00E-04	<1.00E-04	<1.00E-04	0.000898	0.0281	0.333	1.95	6.66	15.6
**5.00**	<1.00E-04	<1.00E-04	<1.00E-04	<1.00E-04	<1.00E-04	0.00182	0.0786	0.842	4.06	11.5
**3.33**	<1.00E-04	<1.00E-04	<1.00E-04	<1.00E-04	<1.00E-04	0.000125	0.0202	0.392	2.61	8.91
**1.67**	<1.00E-04	<1.00E-04	<1.00E-04	<1.00E-04	<1.00E-04	<1.00E-04	0.00668	0.226	1.92	7.48
**0.00**	<1.00E-04	<1.00E-04	<1.00E-04	<1.00E-04	<1.00E-04	<1.00E-04	0.00429	0.187	1.74	7.03

Stage 2 Failure Probability (%)
Fill Count = 5

Fill Count

Composite RSD (%)	1	2	3	4	5	6	7	8	9	10
**15.00**	<1.00E-04	<1.00E-04	<1.00E-04	<1.00E-04	0.000518	0.00188	0.00742	0.0240	0.0700	0.188
**13.33**	<1.00E-04	<1.00E-04	<1.00E-04	<1.00E-04	<1.00E-04	0.000173	0.00139	0.00534	0.0198	0.0632
**11.67**	<1.00E-04	<1.00E-04	<1.00E-04	<1.00E-04	<1.00E-04	<1.00E-04	0.000141	0.000860	0.00564	0.0239
**10.00**	<1.00E-04	<1.00E-04	<1.00E-04	<1.00E-04	<1.00E-04	<1.00E-04	<1.00E-04	<1.00E-04	0.00208	0.00742
**8.33**	<1.00E-04	<1.00E-04	<1.00E-04	<1.00E-04	<1.00E-04	<1.00E-04	<1.00E-04	<1.00E-04	0.000137	0.00138
**6.67**	<1.00E-04	<1.00E-04	<1.00E-04	<1.00E-04	<1.00E-04	<1.00E-04	<1.00E-04	<1.00E-04	<1.00E-04	0.000478
**5.00**	<1.00E-04	<1.00E-04	<1.00E-04	<1.00E-04	<1.00E-04	<1.00E-04	<1.00E-04	<1.00E-04	<1.00E-04	0.000104
**3.33**	<1.00E-04	<1.00E-04	<1.00E-04	<1.00E-04	<1.00E-04	<1.00E-04	<1.00E-04	<1.00E-04	<1.00E-04	<1.00E-04
**1.67**	<1.00E-04	<1.00E-04	<1.00E-04	<1.00E-04	<1.00E-04	<1.00E-04	<1.00E-04	<1.00E-04	<1.00E-04	<1.00E-04
**0.00**	<1.00E-04	<1.00E-04	<1.00E-04	<1.00E-04	<1.00E-04	<1.00E-04	<1.00E-04	<1.00E-04	<1.00E-04	<1.00E-04

Stage 1 Acceptance Value (%)
Fill Count = 5

Fill Count

Composite RSD (%)	1	2	3	4	5	6	7	8	9	10
**15.00**	11	11	11	12	12	13	13	14	15	16
**13.33**	9.9	10	10	11	11	12	12	13	14	15
**11.67**	8.7	8.8	9.1	9.5	10	11	11	12	13	14
**10.00**	7.5	7.6	7.9	8.4	9.0	9.7	11	11	12	13
**8.33**	6.3	6.5	6.8	7.3	8.0	8.8	9.7	11	12	13
**6.67**	5.1	5.3	5.7	6.3	7.1	8.0	9.0	10	11	12
**5.00**	3.9	4.1	4.6	5.4	6.3	7.3	8.4	9.5	11	12
**3.33**	2.6	2.9	3.6	4.6	5.7	6.8	7.9	9.1	10	11
**1.67**	1.3	1.8	2.9	4.1	5.3	6.5	7.6	8.8	10	11
**0.00**	0	1.3	2.6	3.9	5.1	6.3	7.5	8.7	9.9	11

C Confidential

Stage 2 Acceptance Value (%)
Fill Count = 5

Fill Count

Composite RSD (%)	1	2	3	4	5	6	7	8	9	10
**15.00**	9.4	9.5	9.6	9.9	10	11	11	12	13	13
**13.33**	8.4	8.5	8.7	9.0	9.4	9.9	10	11	12	13
**11.67**	7.4	7.5	7.7	8.0	8.5	9.0	9.7	10	11	12
**10.00**	6.3	6.4	6.7	7.1	7.6	8.2	8.9	9.7	10	11
**8.33**	5.3	5.4	5.7	6.2	6.8	7.5	8.2	9.0	9.9	11
**6.67**	4.2	4.4	4.7	5.3	6.0	6.8	7.6	8.5	9.4	10
**5.00**	3.2	3.3	3.8	4.5	5.3	6.2	7.1	8.0	9.0	9.9
**3.33**	2.1	2.4	3.0	3.8	4.7	5.7	6.7	7.7	8.7	9.6
**1.67**	1.1	1.5	2.4	3.3	4.4	5.4	6.4	7.5	8.5	9.5
**0.00**	0	1.1	2.1	3.2	4.2	5.3	6.3	7.4	8.4	9.4

Stage 1 Failure Probability (%)
Fill Count = 10

Fill Count

Composite RSD (%)	1	2	3	4	5	6	7	8	9	10
**15.00**	0.0215	0.0245	0.0410	0.0781	0.173	0.395	0.926	2.00	3.97	7.23
**13.33**	0.000848	0.000972	0.00242	0.00623	0.0197	0.0679	0.234	0.698	1.79	3.99
**11.67**	<1.00E-04	<1.00E-04	<1.00E-04	<1.00E-04	0.00110	0.00688	0.0412	0.199	0.699	1.99
**10.00**	<1.00E-04	<1.00E-04	<1.00E-04	<1.00E-04	<1.00E-04	0.000299	0.00481	0.0411	0.231	0.918
**8.33**	<1.00E-04	<1.00E-04	<1.00E-04	<1.00E-04	<1.00E-04	<1.00E-04	0.000374	0.00686	0.0718	0.399
**6.67**	<1.00E-04	<1.00E-04	<1.00E-04	<1.00E-04	<1.00E-04	<1.00E-04	<1.00E-04	0.00102	0.0197	0.169
**5.00**	<1.00E-04	<1.00E-04	<1.00E-04	<1.00E-04	<1.00E-04	<1.00E-04	<1.00E-04	0.000199	0.00566	0.0776
**3.33**	<1.00E-04	<1.00E-04	<1.00E-04	<1.00E-04	<1.00E-04	<1.00E-04	<1.00E-04	<1.00E-04	0.00167	0.0382
**1.67**	<1.00E-04	<1.00E-04	<1.00E-04	<1.00E-04	<1.00E-04	<1.00E-04	<1.00E-04	<1.00E-04	0.000972	0.0255
**0.00**	<1.00E-04	<1.00E-04	<1.00E-04	<1.00E-04	<1.00E-04	<1.00E-04	<1.00E-04	<1.00E-04	0.000524	0.0224

Stage 2 Failure Probability (%)
Fill Count = 10

Fill Count

Composite RSD (%)	1	2	3	4	5	6	7	8	9	10
**15.00**	<1.00E-04	<1.00E-04	<1.00E-04	<1.00E-04	<1.00E-04	<1.00E-04	<1.00E-04	<1.00E-04	<1.00E-04	<1.00E-04
**13.33**	<1.00E-04	<1.00E-04	<1.00E-04	<1.00E-04	<1.00E-04	<1.00E-04	<1.00E-04	<1.00E-04	<1.00E-04	<1.00E-04
**11.67**	<1.00E-04	<1.00E-04	<1.00E-04	<1.00E-04	<1.00E-04	<1.00E-04	<1.00E-04	<1.00E-04	<1.00E-04	<1.00E-04
**10.00**	<1.00E-04	<1.00E-04	<1.00E-04	<1.00E-04	<1.00E-04	<1.00E-04	<1.00E-04	<1.00E-04	<1.00E-04	<1.00E-04
**8.33**	<1.00E-04	<1.00E-04	<1.00E-04	<1.00E-04	<1.00E-04	<1.00E-04	<1.00E-04	<1.00E-04	<1.00E-04	<1.00E-04
**6.67**	<1.00E-04	<1.00E-04	<1.00E-04	<1.00E-04	<1.00E-04	<1.00E-04	<1.00E-04	<1.00E-04	<1.00E-04	<1.00E-04
**5.00**	<1.00E-04	<1.00E-04	<1.00E-04	<1.00E-04	<1.00E-04	<1.00E-04	<1.00E-04	<1.00E-04	<1.00E-04	<1.00E-04
**3.33**	<1.00E-04	<1.00E-04	<1.00E-04	<1.00E-04	<1.00E-04	<1.00E-04	<1.00E-04	<1.00E-04	<1.00E-04	<1.00E-04
**1.67**	<1.00E-04	<1.00E-04	<1.00E-04	<1.00E-04	<1.00E-04	<1.00E-04	<1.00E-04	<1.00E-04	<1.00E-04	<1.00E-04
**0.00**	<1.00E-04	<1.00E-04	<1.00E-04	<1.00E-04	<1.00E-04	<1.00E-04	<1.00E-04	<1.00E-04	<1.00E-04	<1.00E-04

C Confidential

Stage 1 Acceptance Value (%)
Fill Count = 10

Fill Count

Composite RSD (%)	1	2	3	4	5	6	7	8	9	10
**15.00**	8.0	8.0	8.2	8.4	8.7	9.1	9.5	10	11	11
**13.33**	7.1	7.2	7.3	7.6	7.9	8.4	8.8	9.4	10	11
**11.67**	6.3	6.3	6.5	6.8	7.2	7.7	8.2	8.8	9.4	10
**10.00**	5.4	5.5	5.7	6.0	6.5	7.0	7.6	8.2	8.8	9.5
**8.33**	4.5	4.6	4.9	5.3	5.8	6.3	7.0	7.7	8.4	9.1
**6.67**	3.6	3.7	4.1	4.5	5.1	5.8	6.5	7.2	7.9	8.7
**5.00**	2.7	2.9	3.3	3.9	4.5	5.3	6.0	6.8	7.6	8.4
**3.33**	1.8	2.0	2.6	3.3	4.1	4.9	5.7	6.5	7.3	8.2
**1.67**	0.91	1.3	2.0	2.9	3.7	4.6	5.5	6.3	7.2	8.0
**0.00**	0	0.91	1.8	2.7	3.6	4.5	5.4	6.3	7.1	8.0

Stage 2 Acceptance Value (%)
Fill Count = 10

Fill Count

Composite RSD (%)	1	2	3	4	5	6	7	8	9	10
**15.00**	6.7	6.8	6.9	7.1	7.3	7.7	8.1	8.5	8.9	9.4
**13.33**	6.0	6.0	6.2	6.4	6.7	7.0	7.5	7.9	8.4	8.9
**11.67**	5.2	5.3	5.4	5.7	6.0	6.4	6.9	7.4	7.9	8.5
**10.00**	4.5	4.5	4.7	5.0	5.4	5.8	6.3	6.9	7.5	8.1
**8.33**	3.7	3.8	4.0	4.4	4.8	5.3	5.8	6.4	7.0	7.7
**6.67**	3.0	3.1	3.3	3.7	4.2	4.8	5.4	6.0	6.7	7.3
**5.00**	2.2	2.4	2.7	3.2	3.7	4.4	5.0	5.7	6.4	7.1
**3.33**	1.5	1.7	2.1	2.7	3.3	4.0	4.7	5.4	6.2	6.9
**1.67**	0.75	1.1	1.7	2.4	3.1	3.8	4.5	5.3	6.0	6.8
**0.00**	0	0.75	1.5	2.2	3.0	3.7	4.5	5.2	6.0	6.7

C Confidential

S2.2. Tables of calculated values corresponding to Figure 5 in main manuscript

Stage 1 Failure Probability (%)
Fill Error Probability Per Mini-tablet Filled = 0%

Fill Count

Composite RSD (%)	1	2	3	4	5	6	7	8	9	10
**15.00**	99.9	97.8	91.0	79.4	65.2	50.5	37.2	26.5	18.1	12.1
**13.33**	99.6	94.9	82.2	64.1	45.9	30.5	19.1	11.5	6.61	3.70
**11.67**	98.9	88	65.8	42.0	24.0	12.5	6.09	2.84	1.27	0.543
**10.00**	96.6	72.4	40.3	18.1	7.06	2.48	0.811	0.252	0.0749	0.0221
**8.33**	88.6	43.8	13.6	3.23	0.653	0.117	0.0188	0.00284	0.000449	<1.00E-04
**6.67**	64.2	11.5	1.09	0.0754	0.00476	0.000224	<1.00E-04	<1.00E-04	<1.00E-04	<1.00E-04
**5.00**	18.1	0.250	0.00170	<1.00E-04	<1.00E-04	<1.00E-04	<1.00E-04	<1.00E-04	<1.00E-04	<1.00E-04
**3.33**	0.0766	<1.00E-04	<1.00E-04	<1.00E-04	<1.00E-04	<1.00E-04	<1.00E-04	<1.00E-04	<1.00E-04	<1.00E-04
**1.67**	<1.00E-04	<1.00E-04	<1.00E-04	<1.00E-04	<1.00E-04	<1.00E-04	<1.00E-04	<1.00E-04	<1.00E-04	<1.00E-04
**0.00**	<1.00E-04	<1.00E-04	<1.00E-04	<1.00E-04	<1.00E-04	<1.00E-04	<1.00E-04	<1.00E-04	<1.00E-04	<1.00E-04

Stage 2 Failure Probability (%)
Fill Error Probability Per Mini-tablet Filled = 0%

Fill Count

Composite RSD (%)	1	2	3	4	5	6	7	8	9	10
**15.00**	97.0	41.9	9.54	1.95	0.390	0.0748	0.0181	0.00455	0.000860	0.000218
**13.33**	88.1	19.4	2.68	0.356	0.0430	0.00762	0.000796	<1.00E-04	<1.00E-04	<1.00E-04
**11.67**	64.2	5.88	0.406	0.0309	0.00208	0.000218	<1.00E-04	<1.00E-04	<1.00E-04	<1.00E-04
**10.00**	29.6	0.851	0.0206	0.000646	<1.00E-04	<1.00E-04	<1.00E-04	<1.00E-04	<1.00E-04	<1.00E-04
**8.33**	6.47	0.0323	0.000324	<1.00E-04	<1.00E-04	<1.00E-04	<1.00E-04	<1.00E-04	<1.00E-04	<1.00E-04
**6.67**	0.351	<1.00E-04	<1.00E-04	<1.00E-04	<1.00E-04	<1.00E-04	<1.00E-04	<1.00E-04	<1.00E-04	<1.00E-04
**5.00**	0.000478	<1.00E-04	<1.00E-04	<1.00E-04	<1.00E-04	<1.00E-04	<1.00E-04	<1.00E-04	<1.00E-04	<1.00E-04
**3.33**	<1.00E-04	<1.00E-04	<1.00E-04	<1.00E-04	<1.00E-04	<1.00E-04	<1.00E-04	<1.00E-04	<1.00E-04	<1.00E-04
**1.67**	<1.00E-04	<1.00E-04	<1.00E-04	<1.00E-04	<1.00E-04	<1.00E-04	<1.00E-04	<1.00E-04	<1.00E-04	<1.00E-04
**0.00**	<1.00E-04	<1.00E-04	<1.00E-04	<1.00E-04	<1.00E-04	<1.00E-04	<1.00E-04	<1.00E-04	<1.00E-04	<1.00E-04

Stage 1 Acceptance Value (%)
Fill Error Probability Per Mini-tablet Filled = 0%

Fill Count

Composite RSD (%)	1	2	3	4	5	6	7	8	9	10
**15.00**	38	26	22	19	17	15	14	13	12	12
**13.33**	33	23	19	17	15	14	13	12	11	11
**11.67**	29	20	17	14	13	12	11	10	9.7	9.2
**10.00**	25	18	14	12	11	10	9.5	8.9	8.4	8.0
**8.33**	21	15	12	10	9.3	8.6	7.9	7.5	7.1	6.7
**6.67**	17	12	9.6	8.4	7.5	6.9	6.4	6.0	5.7	5.4
**5.00**	12	8.9	7.3	6.4	5.7	5.3	4.9	4.6	4.3	4.1
**3.33**	8.4	6.0	5.0	4.3	3.9	3.5	3.3	3.0	2.9	2.7
**1.67**	4.3	3.0	2.5	2.2	1.9	1.8	1.6	1.5	1.4	1.4
**0.00**	<1.00E-04	<1.00E-04	<1.00E-04	<1.00E-04	<1.00E-04	<1.00E-04	<1.00E-04	<1.00E-04	<1.00E-04	<1.00E-04

C Confidential

Stage 2 Acceptance Value (%)
Fill Error Probability Per Mini-tablet Filled = 0%

Fill Count

Composite RSD (%)	1	2	3	4	5	6	7	8	9	10
**15.00**	31	22	18	16	14	13	12	11	10	10
**13.33**	28	19	16	14	12	11	11	9.9	9.4	8.9
**11.67**	24	17	14	12	11	10	9.3	8.7	8.2	7.8
**10.00**	21	15	12	10	9.4	8.6	8.0	7.5	7.1	6.7
**8.33**	17	12	10	8.8	7.9	7.2	6.7	6.3	5.9	5.6
**6.67**	14	9.9	8.1	7.1	6.3	5.8	5.4	5.0	4.7	4.5
**5.00**	10	7.5	6.1	5.3	4.8	4.3	4.0	3.8	3.5	3.4
**3.33**	7.1	5.0	4.1	3.5	3.2	2.9	2.7	2.5	2.4	2.2
**1.67**	3.5	2.5	2.0	1.8	1.6	1.4	1.3	1.3	1.2	1.1
**0.00**	<1.00E-04	<1.00E-04	<1.00E-04	<1.00E-04	<1.00E-04	<1.00E-04	<1.00E-04	<1.00E-04	<1.00E-04	<1.00E-04

Stage 1 Failure Probability (%)
Fill Error Probability Per Mini-tablet Filled = 0.0001%

Fill Count

Composite RSD (%)	1	2	3	4	5	6	7	8	9	10
**15.00**	99.9	97.8	91.1	79.5	65.1	50.5	37.1	26.4	18.1	12.1
**13.33**	99.6	94.9	82.1	64.2	45.8	30.5	19.1	11.5	6.62	3.70
**11.67**	98.9	88.00	65.7	42.00	23.9	12.5	6.09	2.84	1.27	0.549
**10.00**	96.6	72.5	40.3	18.1	7.06	2.48	0.813	0.259	0.0782	0.0237
**8.33**	88.7	43.8	13.5	3.24	0.653	0.120	0.0209	0.00434	0.000648	0.000125
**6.67**	64.2	11.5	1.07	0.0755	0.00772	0.00197	0.000823	9.97E-05	<1.00E-06	<1.00E-06
**5.00**	18.1	0.244	0.00396	0.00317	0.00295	0.00115	0.000374	2.49E-05	<1.00E-06	<1.00E-06
**3.33**	0.0759	0.00129	0.00158	0.00297	0.00332	0.000399	<1.00E-06	<1.00E-06	<1.00E-06	<1.00E-06
**1.67**	0.000432	0.000794	0.00208	0.00386	0.00319	7.48E-05	<1.00E-06	<1.00E-06	<1.00E-06	<1.00E-06
**0.00**	0.000860	0.00129	0.00257	0.00279	0.00292	<1.00E-06	<1.00E-06	<1.00E-06	<1.00E-06	<1.00E-06

Stage 2 Failure Probability (%)
Fill Error Probability Per Mini-tablet Filled = 0.0001%

Fill Count

Composite RSD (%)	1	2	3	4	5	6	7	8	9	10
**15.00**	97.1	42.0	9.48	1.93	0.386	0.0756	0.0153	0.00356	0.000696	0.000120
**13.33**	88.1	19.5	2.74	0.350	0.0461	0.00564	0.000927	0.000130	<1.00E-04	<1.00E-04
**11.67**	64.2	5.86	0.419	0.0293	0.00336	0.000239	<1.00E-04	<1.00E-04	<1.00E-04	<1.00E-04
**10.00**	29.5	0.874	0.0267	0.00218	0.000342	<1.00E-04	<1.00E-04	<1.00E-04	<1.00E-04	<1.00E-04
**8.33**	6.50	0.0397	0.00257	0.00148	0.000374	<1.00E-04	<1.00E-04	<1.00E-04	<1.00E-04	<1.00E-04
**6.67**	0.347	0.00168	0.00227	0.00168	0.000133	<1.00E-04	<1.00E-04	<1.00E-04	<1.00E-04	<1.00E-04
**5.00**	0.00106	0.00119	0.00198	0.00129	<1.00E-04	<1.00E-04	<1.00E-04	<1.00E-04	<1.00E-04	<1.00E-04
**3.33**	0.000796	0.00129	0.00158	0.00168	<1.00E-04	<1.00E-04	<1.00E-04	<1.00E-04	<1.00E-04	<1.00E-04
**1.67**	0.000432	0.000794	0.00208	0.00158	<1.00E-04	<1.00E-04	<1.00E-04	<1.00E-04	<1.00E-04	<1.00E-04
**0.00**	0.000860	0.00129	0.00257	<1.00E-04	<1.00E-04	<1.00E-04	<1.00E-04	<1.00E-04	<1.00E-04	<1.00E-04

C Confidential

Stage 1 Acceptance Value (%)
Fill Error Probability Per Mini-tablet Filled = 0.0001%

Fill Count

Composite RSD (%)	1	2	3	4	5	6	7	8	9	10
**15.00**	38	26	22	19	17	15	14	13	12	12
**13.33**	33	23	19	17	15	14.0	13	12	11	11
**11.67**	29	20	17	14	13	12	11	10	9.7	9.2
**10.00**	25	18	14	12	11	10	9.5	8.9	8.4	8
**8.33**	21	15	12	10	9.3	8.6	7.9	7.5	7.1	6.7
**6.67**	17	12	9.6	8.4	7.5	6.9	6.4	6	5.70	5.4
**5.00**	12	8.9	7.3	6.4	5.7	5.3	4.9	4.6	4.3	4.1
**3.33**	8.4	6.0	5.0	4.3	3.9	3.5	3.3	3	2.9	2.7
**1.67**	4.3	3.1	2.5	2.2	1.9	1.8	1.6	1.5	1.4	1.4
**0.00**	0.00073	0.00053	0.00070	0.00056	0.00046	0.00053	0.00061	0.00056	0.00058	0.00065

Stage 2 Acceptance Value (%)
Fill Error Probability Per Mini-tablet Filled = 0.0001%

Fill Count

Composite RSD (%)	1	2	3	4	5	6	7	8	9	10
**15.00**	31	22	18	16	14	13	12	11	10	10
**13.33**	28	19	16	14	12	11	11	9.9	9.4	8.9
**11.67**	24	17	14	12	11	10	9.3	8.7	8.2	7.8
**10.00**	21	15	12	10	9.4	8.6	8	7.5	7.1	6.7
**8.33**	17	12	10	8.8	7.9	7.2	6.7	6.3	5.9	5.6
**6.67**	14	9.9	8.1	7.1	6.3	5.8	5.4	5.0	4.7	4.5
**5.00**	10	7.5	6.1	5.3	4.8	4.3	4.0	3.8	3.5	3.4
**3.33**	7.1	5.0	4.1	3.5	3.2	2.9	2.7	2.5	2.4	2.2
**1.67**	3.5	2.5	2.0	1.8	1.6	1.4	1.3	1.3	1.2	1.1
**0.00**	0.00094	0.00067	0.00083	0.00077	0.00071	0.00078	0.00080	0.00077	0.00078	0.00074

Stage 1 Failure Probability (%)
Fill Error Probability Per Mini-tablet Filled = 0.001%

Fill Count

Composite RSD (%)	1	2	3	4	5	6	7	8	9	10
**15.00**	99.9	97.8	91.0	79.4	65	50.4	37.2	26.4	18.1	12.1
**13.33**	99.6	94.9	82.2	64.2	45.9	30.5	19.2	11.5	6.62	3.71
**11.67**	98.9	87.9	65.7	42.1	24.0	12.5	6.11	2.85	1.29	0.556
**10.00**	96.6	72.4	40.3	18.2	7.06	2.50	0.834	0.268	0.0821	0.0249
**8.33**	88.6	43.8	13.6	3.22	0.675	0.144	0.0336	0.00895	0.00289	0.000773
**6.67**	64.2	11.5	1.11	0.0972	0.0330	0.0190	0.00743	0.00142	0.000349	<1.00E-04
**5.00**	18.2	0.273	0.0233	0.0259	0.0299	0.0129	0.00247	0.000175	<1.00E-04	<1.00E-04
**3.33**	0.0835	0.0134	0.0224	0.0265	0.0285	0.00641	<1.00E-04	<1.00E-04	<1.00E-04	<1.00E-04
**1.67**	0.00702	0.0135	0.0193	0.0269	0.0306	0.000598	<1.00E-04	<1.00E-04	<1.00E-04	<1.00E-04
**0.00**	0.00722	0.0150	0.0200	0.0274	0.0332	<1.00E-04	<1.00E-04	<1.00E-04	<1.00E-04	<1.00E-04

Stage 2 Failure Probability (%)
Fill Error Probability Per Mini-tablet Filled = 0.001%

Fill Count

Composite RSD (%)	1	2	3	4	5	6	7	8	9	10
**15.00**	97.1	42.1	9.51	1.99	0.402	0.0838	0.0155	0.00376	0.00129	0.000218
**13.33**	88.1	19.4	2.75	0.369	0.0515	0.0114	0.00237	0.000370	<1.00E-04	<1.00E-04
**11.67**	64.3	5.89	0.452	0.0516	0.00989	0.00208	0.000310	0.000124	<1.00E-04	<1.00E-04
**10.00**	29.5	0.896	0.0541	0.0171	0.00465	0.000746	<1.00E-04	<1.00E-04	<1.00E-04	<1.00E-04
**8.33**	6.53	0.0615	0.0254	0.0144	0.00317	0.000342	<1.00E-04	<1.00E-04	<1.00E-04	<1.00E-04
**6.67**	0.368	0.0172	0.0218	0.0121	0.00119	<1.00E-04	<1.00E-04	<1.00E-04	<1.00E-04	<1.00E-04
**5.00**	0.0104	0.0140	0.0217	0.0118	0.000597	<1.00E-04	<1.00E-04	<1.00E-04	<1.00E-04	<1.00E-04
**3.33**	0.00791	0.0134	0.0224	0.0112	<1.00E-04	<1.00E-04	<1.00E-04	<1.00E-04	<1.00E-04	<1.00E-04
**1.67**	0.00702	0.0135	0.0193	0.0140	<1.00E-04	<1.00E-04	<1.00E-04	<1.00E-04	<1.00E-04	<1.00E-04
**0.00**	0.00722	0.0150	0.0200	<1.00E-04	<1.00E-04	<1.00E-04	<1.00E-04	<1.00E-04	<1.00E-04	<1.00E-04

Stage 1 Acceptance Value (%)
Fill Error Probability Per Mini-tablet Filled = 0.001%

Fill Count

Composite RSD (%)	1	2	3	4	5	6	7	8	9	10
**15.00**	38	26	22	19	17	15	14	13	12	12
**13.33**	33	23	19	17	15	14	13	12	11	11
**11.67**	29	20	17	14	13	12	11	10	9.7	9.2
**10.00**	25	18	14	12	11	10	9.5	8.9	8.4	8.0
**8.33**	21	15	12	10	9.3	8.6	8.0	7.5	7.1	6.7
**6.67**	17	12	9.6	8.4	7.5	6.9	6.4	6.0	5.7	5.4
**5.00**	12	8.9	7.3	6.4	5.7	5.3	4.9	4.6	4.3	4.1
**3.33**	8.4	6.0	5.0	4.3	3.9	3.5	3.3	3.1	2.9	2.7
**1.67**	4.3	3.1	2.5	2.2	1.9	1.8	1.6	1.5	1.4	1.4
**0.00**	0.00610	0.00620	0.00540	0.00550	0.00520	0.00520	0.00560	0.00560	0.00560	0.00580

Stage 2 Acceptance Value (%)
Fill Error Probability Per Mini-tablet Filled = 0.001%

Fill Count

Composite RSD (%)	1	2	3	4	5	6	7	8	9	10
**15.00**	31	22	18	16	14	13	12	11	10	10
**13.33**	28	19	16	14	12	11	11	9.9	9.4	8.9
**11.67**	24	17	14	12	11	10	9.3	8.7	8.2	7.8
**10.00**	21	15	12	10	9.4	8.6	8.0	7.5	7.1	6.7
**8.33**	17	12	10	8.8	7.9	7.2	6.7	6.3	5.9	5.6
**6.67**	14	9.9	8.1	7.1	6.3	5.8	5.4	5.0	4.7	4.5
**5.00**	10	7.5	6.1	5.3	4.8	4.3	4.0	3.8	3.6	3.4
**3.33**	7.1	5.0	4.1	3.6	3.2	2.9	2.7	2.5	2.4	2.2
**1.67**	3.6	2.5	2.1	1.8	1.6	1.5	1.3	1.3	1.2	1.1
**0.00**	0.00850	0.00740	0.00780	0.00820	0.00810	0.00800	0.00780	0.00780	0.00790	0.00780

Stage 1 Failure Probability (%)
Fill Error Probability Per Mini-tablet Filled = 0.01%

Fill Count

Composite RSD (%)	1	2	3	4	5	6	7	8	9	10
**15.00**	99.9	97.8	91.0	79.5	65.1	50.5	37.4	26.5	18.3	12.2
**13.33**	99.6	94.9	82.2	64.3	46.0	30.7	19.3	11.7	6.81	3.84
**11.67**	98.9	88.0	65.8	42.3	24.2	12.7	6.36	3.02	1.40	0.628
**10.00**	96.6	72.5	40.4	18.4	7.28	2.76	1.02	0.378	0.144	0.0548
**8.33**	88.7	44.0	13.7	3.47	0.938	0.356	0.161	0.0668	0.0233	0.00711
**6.67**	64.3	11.6	1.26	0.333	0.291	0.194	0.0740	0.0186	0.00307	0.000623
**5.00**	18.2	0.378	0.198	0.270	0.282	0.128	0.0215	0.00213	0.000224	0.000183
**3.33**	0.141	0.132	0.198	0.270	0.28	0.0531	0.00238	0.000322	<1.00E-04	<1.00E-04
**1.67**	0.0684	0.140	0.202	0.267	0.296	0.00552	0.00103	<1.00E-04	0.000125	<1.00E-04
**0.00**	0.0700	0.127	0.208	0.263	0.332	0.000818	0.00117	<1.00E-04	<1.00E-04	<1.00E-04

Stage 2 Failure Probability (%)
Fill Error Probability Per Mini-tablet Filled = 0.01%

Fill Count

Composite RSD (%)	1	2	3	4	5	6	7	8	9	10
**15.00**	97.1	42.2	9.94	2.27	0.548	0.141	0.0368	0.00890	0.00227	0.000478
**13.33**	88.2	19.8	3.13	0.647	0.160	0.0423	0.0110	0.00168	0.000478	0.000160
**11.67**	64.3	6.20	0.826	0.255	0.0740	0.0183	0.00425	0.000794	0.000126	<1.00E-04
**10.00**	29.7	1.20	0.351	0.177	0.0474	0.00752	0.000993	0.000171	<1.00E-04	<1.00E-04
**8.33**	6.64	0.287	0.246	0.138	0.0264	0.00267	0.000239	<1.00E-04	<1.00E-04	<1.00E-04
**6.67**	0.497	0.158	0.188	0.128	0.0148	0.000558	<1.00E-04	<1.00E-04	<1.00E-04	<1.00E-04
**5.00**	0.0932	0.129	0.196	0.131	0.00406	<1.00E-04	<1.00E-04	<1.00E-04	<1.00E-04	<1.00E-04
**3.33**	0.0645	0.132	0.198	0.131	0.000299	<1.00E-04	<1.00E-04	<1.00E-04	<1.00E-04	<1.00E-04
**1.67**	0.0684	0.140	0.202	0.128	<1.00E-04	<1.00E-04	<1.00E-04	<1.00E-04	<1.00E-04	<1.00E-04
**0.00**	0.0700	0.127	0.208	<1.00E-04	<1.00E-04	<1.00E-04	<1.00E-04	<1.00E-04	<1.00E-04	<1.00E-04

Stage 1 Acceptance Value (%)
Fill Error Probability Per Mini-tablet Filled = 0.01%

Fill Count

Composite RSD (%)	1	2	3	4	5	6	7	8	9	10
**15.00**	38	26	22	19	17	15	14	13	12	12
**13.33**	33	23	19	17	15	14	13	12	11	11
**11.67**	29	21	17	14	13	12	11	10	9.7	9.3
**10.00**	25	18	14	12	11	10	9.5	8.9	8.4	8.0
**8.33**	21	15	12	10	9.4	8.6	8.0	7.5	7.1	6.7
**6.67**	17	12	9.7	8.4	7.6	6.9	6.5	6.1	5.7	5.4
**5.00**	12	8.9	7.4	6.4	5.8	5.3	4.9	4.6	4.3	4.1
**3.33**	8.4	6.1	5.0	4.3	3.9	3.6	3.3	3.1	2.9	2.8
**1.67**	4.4	3.1	2.5	2.2	2.0	1.8	1.7	1.6	1.5	1.4
**0.00**	0.059	0.053	0.056	0.053	0.052	0.05	0.057	0.057	0.057	0.058

Stage 2 Acceptance Value (%)
Fill Error Probability Per Mini-tablet Filled = 0.01%

Composite RSD (%)	Fill Count									
	1	2	3	4	5	6	7	8	9	10
**15.00**	31	22	18	16	14	13	12	11	11	10
**13.33**	28	20	16	14	12	11	11	9.9	9.4	8.9
**11.67**	24	17	14	12	11	10	9.3	8.7	8.2	7.8
**10.00**	21	15	12	11	9.4	8.6	8.0	7.5	7.1	6.7
**8.33**	17	12	10	8.8	7.9	7.2	6.7	6.3	5.9	5.6
**6.67**	14	10	8.2	7.1	6.4	5.8	5.4	5.0	4.8	4.5
**5.00**	11	7.5	6.2	5.4	4.8	4.4	4.1	3.8	3.6	3.4
**3.33**	7.1	5.1	4.2	3.6	3.2	2.9	2.7	2.6	2.4	2.3
**1.67**	3.6	2.6	2.1	1.8	1.7	1.5	1.4	1.3	1.2	1.2
**0.00**	0.078	0.074	0.080	0.079	0.080	0.079	0.079	0.080	0.079	0.079

Stage 1 Failure Probability (%)
Fill Error Probability Per Mini-tablet Filled = 0.1%

Composite RSD (%)	Fill Count									
	1	2	3	4	5	6	7	8	9	10
**15.00**	99.9	97.8	91.2	79.9	66.1	51.9	39.1	28.4	20.1	13.9
**13.33**	99.6	94.9	82.5	65.0	47.5	32.6	21.5	13.6	8.41	5.10
**11.67**	98.9	88.2	66.4	43.6	26.3	15.1	8.50	4.72	2.59	1.43
**10.00**	96.6	72.8	41.4	20.3	9.79	5.20	2.90	1.57	0.796	0.381
**8.33**	88.7	44.5	15.2	5.72	3.52	2.52	1.47	0.684	0.278	0.103
**6.67**	64.4	12.6	3.03	2.70	2.83	1.90	0.760	0.234	0.0724	0.0269
**5.00**	18.7	1.56	1.99	2.63	2.79	1.29	0.299	0.0771	0.0254	0.0118
**3.33**	0.737	1.32	1.97	2.62	2.77	0.604	0.109	0.0403	0.0115	0.0101
**1.67**	0.669	1.32	1.97	2.63	2.93	0.123	0.0985	0.0128	0.00967	0.0103
**0.00**	0.661	1.33	1.98	2.61	3.29	0.0751	0.103	0.00733	0.00937	0.00947

Stage 2 Failure Probability (%)
Fill Error Probability Per Mini-tablet Filled = 0.1%

Composite RSD (%)	Fill Count									
	1	2	3	4	5	6	7	8	9	10
**15.00**	97.1	44.1	13.5	5.09	1.98	0.723	0.237	0.0735	0.0251	0.00851
**13.33**	88.4	22.5	6.92	3.18	1.20	0.373	0.105	0.0263	0.00821	0.00257
**11.67**	64.8	9.19	4.38	2.32	0.775	0.184	0.0398	0.0105	0.00267	0.00129
**10.00**	30.9	4.05	3.27	1.73	0.458	0.091	0.0158	0.00366	0.000927	0.000498
**8.33**	8.23	2.49	2.38	1.35	0.283	0.0317	0.00495	0.00277	0.00198	0.000324
**6.67**	1.85	1.63	1.98	1.29	0.152	0.00742	0.00396	0.00198	0.000927	0.000299
**5.00**	0.907	1.31	1.98	1.30	0.0433	0.00366	0.00504	0.00346	0.000173	<1.00E-04
**3.33**	0.663	1.32	1.97	1.32	0.00435	0.00346	0.00435	0.00198	<1.00E-04	<1.00E-04
**1.67**	0.669	1.32	1.97	1.31	0.00297	0.00356	0.00633	0.00366	<1.00E-04	<1.00E-06
**0.00**	0.661	1.33	1.98	0.00138	0.00218	0.00287	0.00584	<1.00E-04	<1.00E-04	<1.00E-04

Stage 1 Acceptance Value (%)
Fill Error Probability Per Mini-tablet Filled = 0.1%

Fill Count

Composite RSD (%)	1	2	3	4	5	6	7	8	9	10
**15.00**	38	27	22	19	17	15	14	13	13	12
**13.33**	34	24	19	17	15	14	13	12	11	11
**11.67**	30	21	17	15	13	12	11	11	9.9	9.4
**10.00**	25	18	15	13	11	10	9.7	9.1	8.6	8.2
**8.33**	21	15	12	11	9.6	8.8	8.2	7.7	7.3	7.0
**6.67**	17	12	10	8.7	7.9	7.2	6.7	6.3	6.0	5.7
**5.00**	13	9.3	7.7	6.8	6.1	5.6	5.2	4.9	4.6	4.4
**3.33**	8.9	6.5	5.4	4.7	4.3	3.9	3.7	3.5	3.3	3.1
**1.67**	4.8	3.6	3.0	2.6	2.4	2.2	2.1	2.0	1.9	1.8
**0.00**	0.56	0.55	0.54	0.52	0.52	0.51	0.57	0.56	0.56	0.56

Stage 2 Acceptance Value (%)
Fill Error Probability Per Mini-tablet Filled = 0.1%

Fill Count

Composite RSD (%)	1	2	3	4	5	6	7	8	9	10
**15.00**	31	22	18	16	14	13	12	11	11	10
**13.33**	28	20	16	14	13	12	11	10	9.5	9.1
**11.67**	25	17	14	12	11	10	9.5	8.9	8.4	8.0
**10.00**	21	15	12	11	9.7	8.9	8.2	7.7	7.3	6.9
**8.33**	18	13	10	9.1	8.2	7.5	7.0	6.5	6.2	5.8
**6.67**	14	10	8.6	7.5	6.7	6.1	5.7	5.3	5.0	4.8
**5.00**	11	8.0	6.6	5.8	5.2	4.8	4.4	4.1	3.9	3.7
**3.33**	7.7	5.6	4.7	4.1	3.7	3.4	3.2	3.0	2.8	2.7
**1.67**	4.2	3.2	2.7	2.4	2.2	2.1	1.9	1.8	1.8	1.7
**0.00**	0.77	0.73	0.78	0.78	0.77	0.76	0.76	0.76	0.75	0.75

C Confidential

Stage 1 Failure Probability (%)
Fill Error Probability Per Mini-tablet Filled = 1%

Fill Count

Composite RSD (%)	1	2	3	4	5	6	7	8	9	10
**15.00**	99.9	98.0	92.7	84.2	74.0	63.5	53.6	44.5	36.1	28.9
**13.33**	99.7	95.6	85.4	72.4	59.8	49.0	39.7	31.5	24.6	18.7
**11.67**	99.0	89.5	71.9	55.4	43.7	35.3	28.1	21.6	16.0	11.6
**10.00**	96.8	76.0	51.1	37.0	31.1	26.5	20.8	15.1	10.4	7.02
**8.33**	89.4	50.9	29.2	25.6	26.0	22.2	15.7	10.4	6.66	4.24
**6.67**	66.5	22.6	19.0	23.3	25.1	18.7	11.3	7.04	4.30	2.68
**5.00**	23.4	12.7	18.2	23.3	24.6	14.5	8.09	5.13	2.89	1.89
**3.33**	6.57	12.5	18.1	23.4	24.5	9.41	7.05	3.75	2.19	1.64
**1.67**	6.44	12.6	18.1	23.3	25.7	6.13	7.35	2.18	2.13	1.47
**0.00**	6.49	12.5	18.2	23.4	28.3	5.80	7.53	1.82	2.04	1.17

Stage 2 Failure Probability (%)
Fill Error Probability Per Mini-tablet Filled = 1%

Fill Count

Composite RSD (%)	1	2	3	4	5	6	7	8	9	10
**15.00**	97.6	60.5	42.8	29.5	15.9	7.15	3.06	1.30	0.582	0.280
**13.33**	90.2	44.9	37.4	25.7	12.2	4.68	1.75	0.756	0.360	0.163
**11.67**	70.6	34.4	33.0	21.6	8.80	2.76	1.00	0.477	0.231	0.0962
**10.00**	42.0	27.9	27.2	17.3	5.99	1.51	0.635	0.383	0.176	0.0598
**8.33**	22.2	21.5	21.1	14.6	3.96	0.813	0.523	0.333	0.128	0.0338
**6.67**	14.3	14.9	18.2	14.1	2.20	0.501	0.498	0.320	0.0829	0.0190
**5.00**	8.56	12.6	18.1	13.9	0.876	0.413	0.487	0.298	0.0436	0.00930
**3.33**	6.51	12.5	18.1	13.5	0.350	0.365	0.524	0.290	0.0108	0.0112
**1.67**	6.44	12.6	18.1	12.0	0.326	0.371	0.509	0.285	0.00633	0.00732
**0.00**	6.49	12.5	18.2	0.504	0.343	0.361	0.530	0.00455	0.00682	0.00732

Stage 1 Acceptance Value (%)
Fill Error Probability Per Mini-tablet Filled = 1%

Fill Count

Composite RSD (%)	1	2	3	4	5	6	7	8	9	10
**15.00**	41	29	24	21	19	17	16	15	14	13
**13.33**	37	27	22	19	17	16	14	14	13	12
**11.67**	33	24	20	17	15	14	13	12	12	11
**10.00**	29	21	17	15	14	13	12	11	10	10
**8.33**	25	18	15	13	12	11	10	9.9	9.4	8.9
**6.67**	21	16	13	12	11	9.9	9.3	8.8	8.3	8
**5.00**	17	13	11	10	9.2	8.6	8.1	7.7	7.3	7.0
**3.33**	13	11	9.2	8.3	7.7	7.2	6.9	6.6	6.4	6.2
**1.67**	9.5	8.0	7.2	6.6	6.2	5.9	5.8	5.7	5.5	5.3
**0.00**	5.5	5.3	5.1	4.9	4.8	4.6	4.9	4.8	4.7	4.6

Stage 2 Acceptance Value (%)
Fill Error Probability Per Mini-tablet Filled = 1%

Fill Count

Composite RSD (%)	1	2	3	4	5	6	7	8	9	10
**15.00**	35	25	20	18	16	14	13	13	12	11
**13.33**	31	22	18	16	14	13	12	12	11	10
**11.67**	28	20	17	15	13	12	11	10	9.9	9.4
**10.00**	25	18	15	13	12	11	10	9.5	9	8.5
**8.33**	22	16	13	12	11	9.8	9.1	8.6	8.1	7.7
**6.67**	19	14	12	11	9.5	8.8	8.2	7.7	7.3	6.9
**5.00**	16	12	10	9.3	8.5	7.8	7.3	6.9	6.5	6.2
**3.33**	13	10	9.1	8.2	7.5	7	6.6	6.2	5.9	5.6
**1.67**	10	8.5	7.8	7.1	6.7	6.3	6	5.7	5.4	5.2
**0.00**	7.3	6.7	6.6	6.3	6.1	5.8	5.6	5.3	5.1	5.0

S2.3. Tables of calculated values corresponding to Figure 6 in main manuscript

Stage 1 Failure Probability (%)
Composite RSD = 0%

Fill Count

Log Fill Error Probability Per Mini-tablet Filled (%)	1	2	3	4	5	6	7	8	9	10
**1.00**	49.8	74.3	86.4	92.6	95.9	88.0	92.1	83.9	85.0	79.0
**0.44**	17.1	31.2	42.7	52.1	60.1	28.8	35.1	17.0	18.3	11.6
**-0.11**	5.03	9.85	14.3	18.6	22.7	3.70	4.85	0.985	1.11	0.653
**-0.67**	1.41	2.83	4.24	5.57	6.91	0.337	0.432	0.0452	0.0543	0.0472
**-1.22**	0.394	0.814	1.19	1.59	1.98	0.0272	0.0374	0.00257	0.00344	0.00371
**-1.78**	0.115	0.225	0.324	0.444	0.558	0.00215	0.00252	0.000299	0.000274	0.000374
**-2.33**	0.0273	0.0652	0.0900	0.122	0.156	0.000224	<1.00E-04	<1.00E-04	<1.00E-04	<1.00E-04
**-2.89**	0.00712	0.0147	0.0249	0.0355	0.0427	<1.00E-04	<1.00E-04	<1.00E-04	<1.00E-04	<1.00E-04
**-3.44**	0.00277	0.00504	0.00584	0.00888	0.0121	<1.00E-04	<1.00E-04	<1.00E-04	<1.00E-04	<1.00E-04
**-4.00**	0.000860	0.00129	0.00257	0.00279	0.00292	<1.00E-04	<1.00E-04	<1.00E-04	<1.00E-04	<1.00E-04

C Confidential

Stage 2 Failure Probability (%)
Composite RSD = 0%

Fill Count

Log Fill Error Probability Per Mini-tablet Filled (%)	1	2	3	4	5	6	7	8	9	10
**1.00**	49.8	74.3	86.4	76.1	48.3	50.8	62.0	15.7	11.2	14.0
**0.44**	17.1	31.2	42.7	8.16	3.53	3.64	5.38	0.150	0.161	0.211
**-0.11**	5.03	9.85	14.3	0.228	0.194	0.211	0.302	0.00208	0.00257	0.00396
**-0.67**	1.41	2.83	4.24	0.00762	0.0118	0.0171	0.0205	<1.00E-04	<1.00E-04	<1.00E-04
**-1.22**	0.394	0.814	1.19	0.000597	0.000993	0.00126	0.00168	<1.00E-04	<1.00E-04	<1.00E-04
**-1.78**	0.115	0.225	0.324	5.00E-05	0.000114	<1.00E-04	0.000126	<1.00E-04	<1.00E-04	<1.00E-04
**-2.33**	0.0273	0.0652	0.0900	<1.00E-04	<1.00E-04	<1.00E-04	<1.00E-04	<1.00E-04	<1.00E-04	<1.00E-04
**-2.89**	0.00712	0.0147	0.0249	<1.00E-04	<1.00E-04	<1.00E-04	<1.00E-04	<1.00E-04	<1.00E-04	<1.00E-04
**-3.44**	0.00277	0.00504	0.00584	<1.00E-04	<1.00E-04	<1.00E-04	<1.00E-04	<1.00E-04	<1.00E-04	<1.00E-04
**-4.00**	0.000860	0.00129	0.00257	<1.00E-04	<1.00E-04	<1.00E-04	<1.00E-04	<1.00E-04	<1.00E-04	<1.00E-04

Stage 1 Acceptance Value (%)
Composite RSD = 0%

Fill Count

Log Fill Error Probability Per Mini-tablet Filled (%)	1	2	3	4	5	6	7	8	9	10
**1.00**	47	40	34	30	28	25	24	22	21	20
**0.44**	15	14	13	12	11	11	11	11	10	9.7
**-0.11**	4.3	4.2	4	3.9	3.8	3.7	3.9	3.9	3.8	3.7
**-0.67**	1.2	1.2	1.2	1.1	1.1	1.1	1.2	1.2	1.2	1.2
**-1.22**	0.33	0.34	0.32	0.32	0.31	0.30	0.34	0.34	0.34	0.34
**-1.78**	0.097	0.093	0.088	0.089	0.088	0.085	0.095	0.094	0.095	0.095
**-2.33**	0.023	0.027	0.024	0.024	0.025	0.024	0.027	0.027	0.027	0.027
**-2.89**	0.0060	0.0061	0.0068	0.0071	0.0067	0.0066	0.0075	0.0072	0.0077	0.0075
**-3.44**	0.0023	0.0021	0.0016	0.0018	0.0019	0.0018	0.0020	0.0020	0.0020	0.0020
**-4.00**	0.00073	0.00053	0.00070	0.00056	0.00046	0.00053	0.00061	0.00056	0.00058	0.00065

Stage 2 Acceptance Value (%)
Composite RSD = 0%

Fill Count

Log Fill Error Probability Per Mini-tablet Filled (%)	1	2	3	4	5	6	7	8	9	10
**1.00**	49	36	30	26	24	22	20	19	18	17
**0.44**	18	16	14	13	12	11	10	9.8	9.3	8.9
**-0.11**	5.7	5.3	5.3	5.1	5.0	4.8	4.6	4.5	4.3	4.2
**-0.67**	1.6	1.6	1.6	1.6	1.6	1.6	1.6	1.6	1.5	1.5
**-1.22**	0.46	0.44	0.47	0.47	0.47	0.46	0.47	0.46	0.46	0.46
**-1.78**	0.13	0.12	0.13	0.13	0.13	0.13	0.13	0.13	0.13	0.13
**-2.33**	0.031	0.035	0.036	0.037	0.037	0.037	0.037	0.037	0.037	0.037
**-2.89**	0.0096	0.0093	0.010	0.010	0.010	0.010	0.010	0.010	0.010	0.010
**-3.44**	0.0024	0.0029	0.0026	0.0028	0.0029	0.0028	0.0029	0.0029	0.0029	0.0028
**-4.00**	0.00094	0.00067	0.00083	0.00077	0.00071	0.00078	0.00080	0.00077	0.00078	0.00074

C Confidential

Stage 1 Failure Probability (%)
Composite RSD = 2%

Fill Count

Log Fill Error Probability Per Mini-tablet Filled (%)	1	2	3	4	5	6	7	8	9	10
**1.00**	49.8	74.2	86.4	92.6	94.1	88.2	91.5	85	86.1	81.5
**0.44**	17.1	31.1	42.7	52.2	55.4	29.7	34.1	19.2	19.2	14.4
**-0.11**	5.05	9.79	14.4	18.7	20.2	4.29	4.67	1.42	1.16	0.839
**-0.67**	1.43	2.82	4.19	5.6	6.07	0.539	0.435	0.0842	0.0569	0.0497
**-1.22**	0.400	0.781	1.19	1.59	1.73	0.0819	0.0387	0.00742	0.00277	0.00337
**-1.78**	0.108	0.224	0.330	0.453	0.485	0.019	0.00299	0.000366	0.000150	0.000175
**-2.33**	0.0315	0.0605	0.0916	0.121	0.133	0.00541	0.00028	<1.00E-04	<1.00E-04	<1.00E-04
**-2.89**	0.00989	0.0150	0.0277	0.0352	0.0377	0.00117	<1.00E-04	<1.00E-04	<1.00E-04	<1.00E-04
**-3.44**	0.00227	0.00475	0.00831	0.00900	0.00910	0.000299	<1.00E-04	<1.00E-04	<1.00E-04	<1.00E-04
**-4.00**	0.000860	0.00129	0.00257	0.00251	0.00309	0.000249	<1.00E-04	<1.00E-04	<1.00E-04	<1.00E-04

Stage 2 Failure Probability (%)
Composite RSD = 2%

Fill Count

Log Fill Error Probability Per Mini-tablet Filled (%)	1	2	3	4	5	6	7	8	9	10
**1.00**	49.8	74.2	86.4	89.3	48.2	51.0	61.6	38.0	11.7	14.3
**0.44**	17.1	31.1	42.7	34.7	3.39	3.71	5.29	2.32	0.172	0.223
**-0.11**	5.05	9.79	14.4	9.72	0.178	0.218	0.298	0.171	0.00218	0.00386
**-0.67**	1.43	2.82	4.19	2.80	0.0122	0.0143	0.0222	0.0120	0.000113	<1.00E-04
**-1.22**	0.400	0.781	1.19	0.796	0.000597	0.000794	0.00198	0.00129	<1.00E-04	<1.00E-04
**-1.78**	0.108	0.224	0.330	0.229	<1.00E-04	<1.00E-04	0.000133	<1.00E-04	<1.00E-04	<1.00E-04
**-2.33**	0.0315	0.0605	0.0916	0.0592	<1.00E-04	<1.00E-04	<1.00E-04	<1.00E-04	<1.00E-04	<1.00E-04
**-2.89**	0.00989	0.0150	0.0277	0.0169	<1.00E-04	<1.00E-04	<1.00E-04	<1.00E-04	<1.00E-04	<1.00E-04
**-3.44**	0.00227	0.00475	0.00831	0.00406	<1.00E-04	<1.00E-04	<1.00E-04	<1.00E-04	<1.00E-04	<1.00E-04
**-4.00**	0.000860	0.00129	0.00257	0.00152	<1.00E-04	<1.00E-04	<1.00E-04	<1.00E-04	<1.00E-04	<1.00E-04

Stage 1 Acceptance Value (%)
Composite RSD = 2%

Fill Count

Log Fill Error Probability Per Mini-tablet Filled (%)	1	2	3	4	5	6	7	8	9	10
**1.00**	50	41	35	31	28	26	24	22	21	20
**0.44**	19	16	15	13	13	12	11	11	10	10
**-0.11**	9.2	7.5	6.6	6.0	5.6	5.3	5.2	5.1	4.9	4.7
**-0.67**	6.3	4.7	4.0	3.6	3.3	3.1	2.9	2.8	2.7	2.6
**-1.22**	5.5	4.0	3.3	2.9	2.6	2.4	2.2	2.1	2.0	1.9
**-1.78**	5.2	3.7	3.1	2.7	2.4	2.2	2.0	1.9	1.8	1.7
**-2.33**	5.2	3.7	3.0	2.6	2.3	2.1	2.0	1.9	1.7	1.7
**-2.89**	5.2	3.7	3.0	2.6	2.3	2.1	2.0	1.8	1.7	1.6
**-3.44**	5.1	3.7	3.0	2.6	2.3	2.1	2.0	1.8	1.7	1.6
**-4.00**	5.1	3.7	3.0	2.6	2.3	2.1	2.0	1.8	1.7	1.6

C Confidential

Stage 2 Acceptance Value (%)
Composite RSD = 2%

Fill Count

Log Fill Error Probability Per Mini-tablet Filled (%)	1	2	3	4	5	6	7	8	9	10
**1.00**	50	37	30	26	24	22	20	19	18	17
**0.44**	21	17	15	13	12	11	11	9.9	9.4	9.0
**-0.11**	9.4	7.6	6.9	6.3	5.9	5.6	5.3	5.0	4.8	4.6
**-0.67**	5.7	4.4	3.8	3.4	3.2	3.0	2.8	2.7	2.6	2.5
**-1.22**	4.7	3.4	2.8	2.5	2.3	2.1	2.0	1.8	1.8	1.7
**-1.78**	4.4	3.1	2.6	2.2	2.0	1.8	1.7	1.6	1.5	1.4
**-2.33**	4.3	3.0	2.5	2.2	1.9	1.8	1.6	1.5	1.4	1.4
**-2.89**	4.3	3.0	2.5	2.1	1.9	1.7	1.6	1.5	1.4	1.4
**-3.44**	4.3	3.0	2.5	2.1	1.9	1.7	1.6	1.5	1.4	1.3
**-4.00**	4.3	3.0	2.5	2.1	1.9	1.7	1.6	1.5	1.4	1.3

Stage 1 Failure Probability (%)
Composite RSD = 4%

Fill Count

Log Fill Error Probability Per Mini-tablet Filled (%)	1	2	3	4	5	6	7	8	9	10
**1.00**	50.8	74.2	86.4	92.7	93.7	90.0	90.5	87.4	85.6	82.1
**0.44**	18.6	31.1	42.7	52.2	54.3	36.7	33.0	25.0	19.5	15.7
**-0.11**	6.89	9.77	14.4	18.7	19.6	8.47	4.69	2.67	1.33	0.949
**-0.67**	3.28	2.83	4.24	5.59	5.91	1.98	0.500	0.222	0.0746	0.0520
**-1.22**	2.27	0.795	1.19	1.57	1.70	0.513	0.0655	0.0189	0.00513	0.00362
**-1.78**	1.99	0.225	0.332	0.448	0.474	0.141	0.0117	0.00167	0.000249	0.000175
**-2.33**	1.94	0.0613	0.0948	0.127	0.135	0.0390	0.00297	0.000199	<1.00E-04	<1.00E-04
**-2.89**	1.88	0.0169	0.0269	0.0365	0.0375	0.0107	0.000848	<1.00E-04	<1.00E-04	<1.00E-04
**-3.44**	1.87	0.00603	0.00831	0.00900	0.00992	0.00334	0.000249	<1.00E-04	<1.00E-04	<1.00E-04
**-4.00**	1.88	0.00247	0.00257	0.00251	0.00287	0.000997	<1.00E-04	<1.00E-04	<1.00E-04	<1.00E-04

Stage 2 Failure Probability (%)
Composite RSD = 4%

Fill Count

Log Fill Error Probability Per Mini-tablet Filled (%)	1	2	3	4	5	6	7	8	9	10
**1.00**	50.5	74.2	86.4	90.0	50.5	51.8	59.8	41.1	14.0	14.3
**0.44**	17.6	31.1	42.7	38.3	3.86	4.05	5.08	2.74	0.287	0.231
**-0.11**	5.26	9.77	14.4	10.6	0.272	0.235	0.297	0.179	0.0120	0.00445
**-0.67**	1.49	2.83	4.24	2.85	0.0351	0.0156	0.0201	0.0145	0.000696	<1.00E-04
**-1.22**	0.406	0.794	1.19	0.780	0.00851	0.000993	0.00208	0.00138	<1.00E-04	<1.00E-04
**-1.78**	0.116	0.224	0.332	0.219	0.00218	<1.00E-04	<1.00E-04	<1.00E-04	<1.00E-04	<1.00E-04
**-2.33**	0.0318	0.0603	0.0948	0.0627	0.000597	<1.00E-04	<1.00E-04	<1.00E-04	<1.00E-04	<1.00E-04
**-2.89**	0.00861	0.0159	0.0269	0.0180	0.000239	<1.00E-04	<1.00E-04	<1.00E-04	<1.00E-04	<1.00E-04
**-3.44**	0.00227	0.00475	0.00831	0.00406	<1.00E-04	<1.00E-04	<1.00E-04	<1.00E-04	<1.00E-04	<1.00E-04
**-4.00**	0.000860	0.00129	0.00247	0.00146	<1.00E-04	<1.00E-04	<1.00E-04	<1.00E-04	<1.00E-04	<1.00E-04

C Confidential

Stage 1 Acceptance Value (%)
Composite RSD = 4%

Fill Count

Log Fill Error Probability Per Mini-tablet Filled (%)	1	2	3	4	5	6	7	8	9	10
**1.00**	53	42	36	31	28	26	24	23	21	20
**0.44**	23	19	17	15	14	13	12	12	11	11
**-0.11**	14	11	9.2	8.2	7.5	7.0	6.6	6.3	6.1	5.9
**-0.67**	11	8.2	6.8	6.0	5.5	5.0	4.7	4.5	4.2	4.1
**-1.22**	10	7.5	6.2	5.4	4.9	4.4	4.1	3.9	3.7	3.5
**-1.78**	10	7.3	6.0	5.2	4.7	4.3	4.0	3.7	3.5	3.3
**-2.33**	10	7.2	5.9	5.2	4.6	4.2	3.9	3.7	3.5	3.3
**-2.89**	10	7.2	5.9	5.2	4.6	4.2	3.9	3.7	3.5	3.3
**-3.44**	10	7.2	5.9	5.1	4.6	4.2	3.9	3.7	3.4	3.3
**-4.00**	10	7.2	5.9	5.1	4.6	4.2	3.9	3.7	3.4	3.3

Stage 2 Acceptance Value (%)
Composite RSD = 4%

Fill Count

Log Fill Error Probability Per Mini-tablet Filled (%)	1	2	3	4	5	6	7	8	9	10
**1.00**	51	37	31	27	24	22	20	19	18	17
**0.44**	24	18	16	14	13	12	11	10	9.7	9.3
**-0.11**	13	10	8.6	7.8	7.1	6.6	6.2	5.8	5.5	5.3
**-0.67**	9.8	7.2	6	5.3	4.8	4.4	4.1	3.9	3.7	3.5
**-1.22**	8.8	6.3	5.2	4.6	4.1	3.8	3.5	3.3	3.1	2.9
**-1.78**	8.6	6.1	5.0	4.3	3.9	3.6	3.3	3.1	2.9	2.8
**-2.33**	8.5	6.0	4.9	4.3	3.8	3.5	3.2	3.0	2.9	2.7
**-2.89**	8.5	6.0	4.9	4.3	3.8	3.5	3.2	3.0	2.8	2.7
**-3.44**	8.5	6.0	4.9	4.3	3.8	3.5	3.2	3.0	2.8	2.7
**-4.00**	8.5	6.0	4.9	4.3	3.8	3.5	3.2	3.0	2.8	2.7

Stage 1 Failure Probability (%)
Composite RSD = 6%

Fill Count

Log Fill Error Probability Per Mini-tablet Filled (%)	1	2	3	4	5	6	7	8	9	10
**1.00**	73.4	75.3	86.4	92.5	93.9	91.9	90.9	88.9	86.4	83.3
**0.44**	56.0	33.9	42.7	52.0	54.7	44.5	36.1	29.4	23.0	18.0
**-0.11**	49.6	13.4	14.5	18.5	19.9	13.2	6.97	3.98	2.21	1.30
**-0.67**	47.8	6.69	4.37	5.57	6.01	3.59	1.35	0.457	0.187	0.0870
**-1.22**	47.1	4.74	1.37	1.58	1.70	0.999	0.323	0.0713	0.0185	0.00649
**-1.78**	47.0	4.19	0.501	0.443	0.478	0.277	0.0830	0.0160	0.00254	0.000648
**-2.33**	47.0	4.02	0.253	0.127	0.132	0.0796	0.0240	0.00414	0.000648	0.000199
**-2.89**	47.0	4.04	0.198	0.0402	0.0342	0.0222	0.00723	0.00102	<1.00E-04	<1.00E-04
**-3.44**	46.9	3.97	0.187	0.0133	0.0104	0.00566	0.00194	0.000299	<1.00E-04	<1.00E-04
**-4.00**	47.0	4.01	0.173	0.00807	0.00294	0.00145	0.000524	<1.00E-04	<1.00E-04	<1.00E-04

Stage 2 Failure Probability (%)
Composite RSD = 6%

Fill Count

Log Fill Error Probability Per Mini-tablet Filled (%)	1	2	3	4	5	6	7	8	9	10
**1.00**	67.5	75.2	86.4	90.2	58.4	53.1	58.2	43.2	19.5	15.0
**0.44**	29.2	32.6	42.6	39.5	7.70	4.47	4.95	3.01	0.708	0.259
**-0.11**	9.42	10.5	14.2	10.7	1.09	0.267	0.293	0.175	0.0379	0.00653
**-0.67**	2.80	3.05	4.17	2.83	0.235	0.0179	0.0202	0.0149	0.00297	0.000432
**-1.22**	0.814	0.854	1.19	0.762	0.0612	0.00227	0.00168	0.000927	0.000184	<1.00E-04
**-1.78**	0.262	0.232	0.329	0.208	0.0175	0.000299	0.000120	<1.00E-04	<1.00E-04	<1.00E-04
**-2.33**	0.113	0.0684	0.0885	0.0599	0.00465	0.000153	<1.00E-04	<1.00E-04	<1.00E-04	<1.00E-04
**-2.89**	0.0650	0.0204	0.0271	0.0174	0.00119	<1.00E-04	<1.00E-04	<1.00E-04	<1.00E-04	<1.00E-04
**-3.44**	0.0604	0.00613	0.00801	0.00475	0.000342	<1.00E-04	<1.00E-04	<1.00E-04	<1.00E-04	<1.00E-04
**-4.00**	0.0494	0.00129	0.00178	0.000927	0.000109	<1.00E-04	<1.00E-04	<1.00E-04	<1.00E-04	<1.00E-04

Stage 1 Acceptance Value (%)
Composite RSD = 6%

Fill Count

Log Fill Error Probability Per Mini-tablet Filled (%)	1	2	3	4	5	6	7	8	9	10
**1.00**	55	43	36	32	29	26	25	23	22	21
**0.44**	27	22	18	16	15	14	13	12	12	11
**-0.11**	18	14	12	10	9.4	8.7	8.2	7.7	7.3	7.0
**-0.67**	16	11	9.5	8.4	7.6	7	6.5	6.1	5.8	5.5
**-1.22**	15	11	8.9	7.8	7	6.5	6	5.6	5.3	5.1
**-1.78**	15	11	8.8	7.6	6.9	6.3	5.9	5.5	5.2	4.9
**-2.33**	15	11	8.7	7.6	6.8	6.3	5.8	5.5	5.2	4.9
**-2.89**	15	11	8.7	7.6	6.8	6.3	5.8	5.5	5.2	4.9
**-3.44**	15	11	8.7	7.6	6.8	6.3	5.8	5.4	5.1	4.9
**-4.00**	15	11	8.7	7.6	6.8	6.3	5.8	5.4	5.1	4.9

Stage 2 Acceptance Value (%)
Composite RSD = 6%

Fill Count

Log Fill Error Probability Per Mini-tablet Filled (%)	1	2	3	4	5	6	7	8	9	10
**1.00**	52	38	31	27	24	22	21	19	18	17
**0.44**	26	20	17	15	13	12	12	11	10	9.8
**-0.11**	17	12	10	9.3	8.4	7.8	7.2	6.8	6.4	6.1
**-0.67**	14	10	8.3	7.2	6.5	6	5.6	5.2	4.9	4.7
**-1.22**	13	9.2	7.6	6.6	5.9	5.4	5	4.7	4.5	4.2
**-1.78**	13	9.0	7.4	6.4	5.8	5.3	4.9	4.6	4.3	4.1
**-2.33**	13	9.0	7.4	6.4	5.7	5.2	4.8	4.5	4.3	4.1
**-2.89**	13	9.0	7.4	6.4	5.7	5.2	4.8	4.5	4.3	4.0
**-3.44**	13	9.0	7.3	6.4	5.7	5.2	4.8	4.5	4.3	4.0
**-4.00**	13	9.0	7.3	6.4	5.7	5.2	4.8	4.5	4.3	4.0

Stage 1 Failure Probability (%)
Composite RSD = 8%

Fill Count

Log Fill Error Probability Per Mini-tablet Filled (%)	1	2	3	4	5	6	7	8	9	10
**1.00**	92.7	83.6	87.6	92.6	94.1	93.3	92.1	90.4	88.0	85.0
**0.44**	88.1	56.5	48.2	52.7	55.7	50.6	42.4	35.0	28.3	22.5
**-0.11**	86.3	43.0	22.6	20.0	20.5	17.0	11.3	6.80	4.02	2.40
**-0.67**	85.8	38.7	13.3	7.31	6.44	4.98	2.89	1.35	0.575	0.250
**-1.22**	85.6	37.4	10.7	3.45	2.07	1.43	0.783	0.330	0.119	0.0393
**-1.78**	85.6	36.9	9.83	2.33	0.802	0.446	0.219	0.086	0.0284	0.00718
**-2.33**	85.6	36.9	9.62	2.00	0.448	0.160	0.0675	0.0257	0.00777	0.00189
**-2.89**	85.6	36.8	9.65	1.94	0.346	0.0794	0.0236	0.00733	0.00212	0.000673
**-3.44**	85.6	36.8	9.57	1.92	0.334	0.0573	0.0114	0.00309	0.000798	0.000175
**-4.00**	85.6	36.8	9.62	1.89	0.322	0.0500	0.00815	0.00157	0.000224	0.000199

Stage 2 Failure Probability (%)
Composite RSD = 8%

Fill Count

Log Fill Error Probability Per Mini-tablet Filled (%)	1	2	3	4	5	6	7	8	9	10
**1.00**	82.5	83	87.6	90.5	68.6	55.6	56.7	44.8	25.4	16.9
**0.44**	41.6	44.4	45.7	40.5	14.2	5.56	5.1	3.32	1.23	0.426
**-0.11**	16.6	16	16	11	2.58	0.453	0.288	0.197	0.0642	0.0171
**-0.67**	7.8	4.84	4.75	2.91	0.579	0.0714	0.0207	0.0138	0.00673	0.00138
**-1.22**	5.27	1.39	1.36	0.788	0.157	0.0135	0.00237	0.00086	0.000646	<1.00E-04
**-1.78**	4.48	0.403	0.391	0.22	0.0409	0.00396	0.000288	<1.00E-04	<1.00E-04	<1.00E-04
**-2.33**	4.3	0.119	0.106	0.0544	0.0114	0.000696	<1.00E-04	<1.00E-04	<1.00E-04	<1.00E-04
**-2.89**	4.21	0.0474	0.0276	0.0178	0.00425	0.000239	<1.00E-04	<1.00E-04	<1.00E-04	<1.00E-04
**-3.44**	4.21	0.0218	0.00752	0.00425	0.00148	<1.00E-04	<1.00E-04	<1.00E-04	<1.00E-04	<1.00E-04
**-4.00**	4.19	0.0166	0.00218	0.000927	0.000218	<1.00E-04	<1.00E-04	<1.00E-04	<1.00E-04	<1.00E-04

Stage 1 Acceptance Value (%)
Composite RSD = 8%

Fill Count

Log Fill Error Probability Per Mini-tablet Filled (%)	1	2	3	4	5	6	7	8	9	10
**1.00**	58	45	37	33	29	27	25	23	22	21
**0.44**	32	24	20	18	16	15	14	13	13	12
**-0.11**	23	17	14	12	11	10	9.7	9.1	8.7	8.3
**-0.67**	21	15	12	11	9.6	8.8	8.2	7.7	7.3	7.0
**-1.22**	20	14	12	10	9.2	8.4	7.8	7.3	6.9	6.6
**-1.78**	20	14	12	10	9.0	8.3	7.7	7.2	6.8	6.5
**-2.33**	20	14	12	10	9.0	8.2	7.7	7.2	6.8	6.5
**-2.89**	20	14	11	10	9.0	8.2	7.6	7.2	6.8	6.5
**-3.44**	20	14	11	10	9.0	8.2	7.6	7.2	6.8	6.5
**-4.00**	20	14	11	10	9.0	8.2	7.6	7.2	6.8	6.5

Stage 2 Acceptance Value (%)
Composite RSD = 8%

Fill Count

Log Fill Error Probability Per Mini-tablet Filled (%)	1	2	3	4	5	6	7	8	9	10
**1.00**	54	39	32	28	25	23	21	20	19	18
**0.44**	29	22	18	16	14	13	12	12	11	10
**-0.11**	20	15	12	11	9.8	9.0	8.4	7.9	7.5	7.1
**-0.67**	18	13	11	9.2	8.3	7.6	7.0	6.6	6.2	5.9
**-1.22**	17	12	9.9	8.7	7.8	7.1	6.6	6.2	5.8	5.5
**-1.78**	17	12	9.8	8.5	7.6	7	6.5	6.1	5.7	5.4
**-2.33**	17	12	9.7	8.5	7.6	6.9	6.4	6.0	5.7	5.4
**-2.89**	17	12	9.7	8.5	7.6	6.9	6.4	6.0	5.7	5.4
**-3.44**	17	12	9.7	8.5	7.6	6.9	6.4	6.0	5.7	5.4
**-4.00**	17	12	9.7	8.5	7.6	6.9	6.4	6.0	5.7	5.4

Stage 1 Failure Probability (%)
Composite RSD = 10%

Fill Count

Log Fill Error Probability Per Mini-tablet Filled (%)	1	2	3	4	5	6	7	8	9	10
**1.00**	98.3	92.9	91.8	93.7	94.9	94.5	93.4	91.9	89.7	87.2
**0.44**	97.2	81.1	65.8	60.5	59.4	55.9	49.7	42.4	35.3	29.1
**-0.11**	96.8	75.2	48.9	33.1	26.3	21.5	16.4	11.5	7.59	4.88
**-0.67**	96.6	73.2	42.8	22.7	12.9	8.17	5.22	3.14	1.74	0.918
**-1.22**	96.6	72.7	41.0	19.4	8.71	4.11	2.07	1.04	0.498	0.228
**-1.78**	96.6	72.5	40.6	18.5	7.52	2.96	1.17	0.469	0.191	0.0772
**-2.33**	96.6	72.5	40.3	18.2	7.18	2.59	0.908	0.313	0.108	0.037
**-2.89**	96.6	72.5	40.3	18.2	7.09	2.49	0.843	0.269	0.0827	0.0272
**-3.44**	96.6	72.4	40.3	18.1	7.05	2.48	0.821	0.264	0.0781	0.0230
**-4.00**	96.6	72.4	40.3	18.1	7.05	2.49	0.814	0.256	0.0766	0.0228

Stage 2 Failure Probability (%)
Composite RSD = 10%

Fill Count

Log Fill Error Probability Per Mini-tablet Filled (%)	1	2	3	4	5	6	7	8	9	10
**1.00**	89.8	91.7	91.6	91.7	76.8	60.4	56.5	46.1	30.4	20.4
**0.44**	58.9	57.6	56.5	44.7	21.0	8.25	5.62	3.69	1.79	0.746
**-0.11**	39.4	22.6	22.0	13.4	4.42	1.03	0.396	0.224	0.0996	0.0346
**-0.67**	32.4	7.54	6.85	3.74	1.04	0.197	0.0470	0.0191	0.00732	0.00287
**-1.22**	30.4	2.75	1.99	1.02	0.268	0.0500	0.00791	0.00178	0.000796	0.000266
**-1.78**	29.7	1.40	0.576	0.293	0.0788	0.0119	0.00188	0.000288	<1.00E-04	<1.00E-04
**-2.33**	29.6	1.04	0.174	0.0813	0.0193	0.00336	0.000746	<1.00E-04	<1.00E-04	<1.00E-04
**-2.89**	29.7	0.913	0.0592	0.0220	0.00554	0.000993	0.000160	<1.00E-04	<1.00E-04	<1.00E-04
**-3.44**	29.5	0.876	0.0382	0.00910	0.00148	0.000324	<1.00E-04	<1.00E-04	<1.00E-04	<1.00E-04
**-4.00**	29.6	0.863	0.0255	0.00148	0.000427	0.000133	<1.00E-04	<1.00E-04	<1.00E-04	<1.00E-04

C Confidential

Stage 1 Acceptance Value (%)
Composite RSD = 10%

Log Fill Error Probability Per Mini-tablet Filled (%) / **Fill Count**

	**1**	**2**	**3**	**4**	**5**	**6**	**7**	**8**	**9**	**10**
**1.00**	61	46	39	34	30	28	26	24	23	22
**0.44**	36	27	23	20	18	16	15	14	14	13
**-0.11**	28	20	17	15	13	12	11	11	10	9.6
**-0.67**	26	18	15	13	12	11	10	9.4	8.9	8.4
**-1.22**	25	18	15	13	11	10	9.6	9.0	8.5	8.1
**-1.78**	25	18	14	12	11	10	9.5	8.9	8.4	8.0
**-2.33**	25	18	14	12	11	10	9.5	8.9	8.4	8.0
**-2.89**	25	18	14	12	11	10	9.5	8.9	8.4	8.0
**-3.44**	25	18	14	12	11	10	9.5	8.9	8.4	8.0
**-4.00**	25	18	14	12	11	10	9.5	8.9	8.4	8.0

Stage 2 Acceptance Value (%)
Composite RSD = 10%

Log Fill Error Probability Per Mini-tablet Filled (%) / **Fill Count**

	**1**	**2**	**3**	**4**	**5**	**6**	**7**	**8**	**9**	**10**
**1.00**	55	40	33	28	25	23	22	20	19	18
**0.44**	32	24	20	17	15	14	13	12	12	11
**-0.11**	24	17	14	13	11	10	9.7	9.1	8.6	8.2
**-0.67**	22	15	13	11	10	9.1	8.5	8.0	7.5	7.1
**-1.22**	21	15	12	11	9.6	8.8	8.1	7.6	7.2	6.8
**-1.78**	21	15	12	11	9.5	8.7	8.0	7.5	7.1	6.8
**-2.33**	21	15	12	11	9.4	8.6	8.0	7.5	7.1	6.7
**-2.89**	21	15	12	10	9.4	8.6	8.0	7.5	7.1	6.7
**-3.44**	21	15	12	10	9.4	8.6	8.0	7.5	7.1	6.7
**-4.00**	21	15	12	10	9.4	8.6	8.0	7.5	7.1	6.7

S3. Discussion

The following example use cases are provided in addition to those in the main manuscript text.

S3.1. Use Case S1

A scientist wishes to know the maximum acceptable weight RSD allowed to ensure probability of CU failure will be below 0.0001% for a product having a fill count of 10 mini-tablets. The fill error probability is estimated to be 0.1% experimentally and potency RSD is measured experimentally to be 5.0%.

- Weight RSD = ?
- Potency RSD = 5.0%
- Fill error probability = 0.1%
- Fill count = 10
- Probability of failing CU = <0.0001%

Step 1: Determine composite RSD corresponding to 0.0001% probability of failing CU for a 10-count product using the model prediction for 0.1% fill error probability.

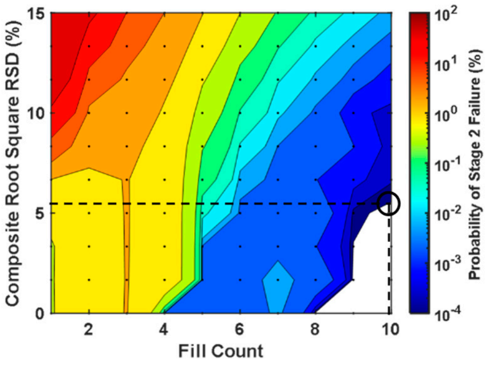

Follow the horizontal axis to the desired fill count of 10 and trace a line up to the point where the probability of failing CU is 0.0001% (the boundary between blue and white region). Then trace a line to the vertical axis to read the composite RSD value (approximately 5.1%) corresponding to this point.

Step 2: Calculate the maximum acceptable weight RSD by rearranging the equation for composite RSD:

$$Composite\ RSD = \sqrt{(Weight\ RSD)^2 + (Potency\ RSD)^2}$$

After rearranging for Weight RSD:

$$Weight\ RSD\ = \sqrt{(Composite\ RSD)^2 - (Potency\ RSD)^2}$$

$$Weight\ RSD\ = \sqrt{5.1\%^2 - 5.0\%^2} = 7.1\%$$

Conclusion: The maximum acceptable weight RSD is 7.1%. Assuming the weight is well controlled for this product, the risk of failing CU is low.

S3.2. Use Case S2

A scientist wishes to predict the expected AV from experimentally measured potency RSD and weight RSD prior to receiving experimental CU results for a recently manufactured batch, since the results may take some time to arrive. The fill error probability was 0%, since the batch was hand-filled with double visual inspection. The product uses a fill count of 1.

- Weight RSD = 5.0%
- Potency RSD = 2.5%
- Fill error probability = 0%
- Fill count = 1
- Expected AV value = ?

Step 1: Determine the AV for a 1-count fill with weight RSD of 5.0% and potency RSD of 2.5% from Figure 4. Draw a vertical line intersecting the horizontal axis at potency RSD equal to 2.5% and a horizontal line intersecting the vertical axis at potency RSD equal to 5.0%. Read the color bar at the intersection of these lines to determine AV value.

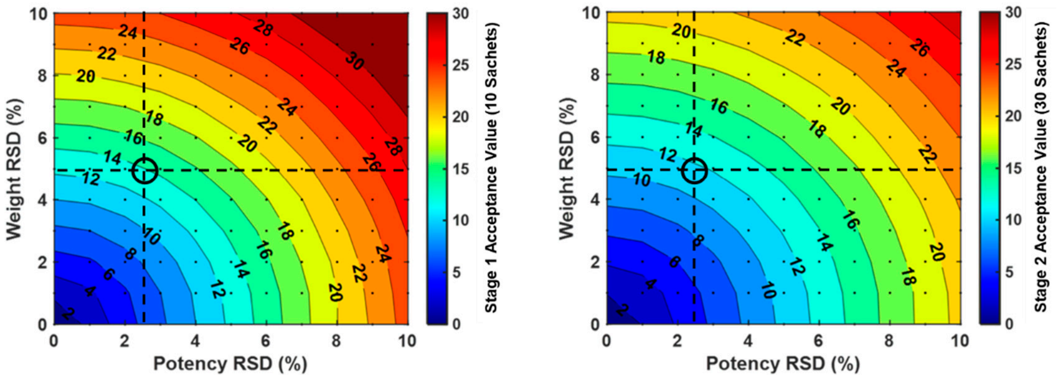

Conclusion: The plots from Figure 4 indicate the mean stage 1 AV is 14 and the mean stage 2 AV is approximately 12. Note, the plots from Figure 4 for a 1-count product are applicable to monolithic tablets as well since the computations are identical.

S3.3. Use Case S3

A scientist wishes to know the maximum acceptable fill error probability during the sachet filling process to achieve a desired target CU failure risk. They would like to communicate this specification to the process engineers operating equipment.

Problem: Determine the maximum allowable fill error probability for a product with the following specifications:

- Weight RSD = 3.0%
- Potency RSD = 3.0%
- Fill error probability = ?
- Fill count = 6
- Allowed probability of failing CU = 0.01%

Solution:
Step 1: Calculate the composite RSD:

$$Composite\ RSD = \sqrt{(Weight\ RSD)^2 + (Potency\ RSD)^2}$$
$$Composite\ RSD = \sqrt{3.0\%^2 + 3.0\%^2} = 4.2\%$$

Step 2: Determine the maximum allowable fill error probability using the plot from Figure 7 for a 4% composite RSD product with a 6-count sachet fill and acceptable content uniformity failure probability of 0.01%:

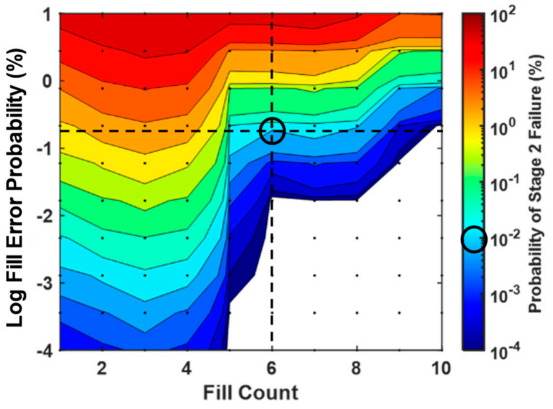

From the plot above, the log-transformed fill error probability corresponding to a 0.01% maximum content uniformity failure probability is approximately -0.8. This results in a log fill error probability of -0.8 which equals a fill error probability of of approximately $10^{-0.8}$ = 0.16% probability per mini-tablet.

Conclusion: The maximum acceptable fill error probability corresponding to the desired content uniformity failure probability of 0.01% is approximately 0.16% (16 errors per 10,000 mini-tablets filled). On average this equates to 16 defective sachets allowed per 1,667 produced (since 10,000 granules will fill 1,667 sachets in this case).

C Confidential

References

1. L. Wasserman, *All of statistics: a concise course in statistical inference* (Vol. 26). Springer.

2. R. Ware and F. Lad, Approximating the distribution for sums of products of normal variables, *Univ. Canterbury, England, Tech. Rep. UCDMS* **15** (2003) 2003.

C Confidential
